# Copy number variation underlies complex phenotypes in domestic dog breeds and other canids

**DOI:** 10.1101/gr.266049.120

**Published:** 2021-05

**Authors:** Aitor Serres-Armero, Brian W. Davis, Inna S. Povolotskaya, Carlos Morcillo-Suarez, Jocelyn Plassais, David Juan, Elaine A. Ostrander, Tomas Marques-Bonet

**Affiliations:** 1 IBE, Institut de Biologia Evolutiva (Universitat Pompeu Fabra/CSIC), Ciencies Experimentals i de la Salut, Barcelona 08003, Spain;; 2 Cancer Genetics and Comparative Genomics Branch, National Human Genome Research Institute, National Institutes of Health, Bethesda, Maryland 20892, USA;; 3 Department of Veterinary Integrative Biosciences, College of Veterinary Medicine, Texas A&M University, College Station, Texas 77843, USA;; 4 Veltischev Research and Clinical Institute for Pediatrics of the Pirogov Russian National Research Medical University, Moscow 117997, Russia;; 5 CNAG-CRG, Centre for Genomic Regulation (CRG), The Barcelona Institute of Science and Technology (BIST), Barcelona 08028, Spain;; 6 Institucio Catalana de Recerca i Estudis Avançats (ICREA), Barcelona, Catalonia 08010, Spain;; 7 Institut Català de Paleontologia Miquel Crusafont, Universitat Autònoma de Barcelona, Cerdanyola del Vallès, Catalonia 08201, Spain

## Abstract

Extreme phenotypic diversity, a history of artificial selection, and socioeconomic value make domestic dog breeds a compelling subject for genomic research. Copy number variation (CNV) is known to account for a significant part of inter-individual genomic diversity in other systems. However, a comprehensive genome-wide study of structural variation as it relates to breed-specific phenotypes is lacking. We have generated whole genome CNV maps for more than 300 canids. Our data set extends the canine structural variation landscape to more than 100 dog breeds, including novel variants that cannot be assessed using microarray technologies. We have taken advantage of this data set to perform the first CNV-based genome-wide association study (GWAS) in canids. We identify 96 loci that display copy number differences across breeds, which are statistically associated with a previously compiled set of breed-specific morphometrics and disease susceptibilities. Among these, we highlight the discovery of a long-range interaction involving a CNV near *MED13L* and *TBX3*, which could influence breed standard height. Integration of the CNVs with chromatin interactions, long noncoding RNA expression, and single nucleotide variation highlights a subset of specific loci and genes with potential functional relevance and the prospect to explain trait variation between dog breeds.

Dogs have been the subject of intense study over many decades (Vilà et al. 1999; Ostrander and Wayne 2005; Freedman et al. 2014; Ostrander et al. 2019), providing valuable insight into human history, disease, and evolution (Coelho et al. 2018; Ní Leathlobhair et al. 2018; Wang et al. 2019). Much has been learned about canines through traditional approaches, including genotype studies with microsatellites (Irion 2003), single nucleotide polymorphisms (SNPs) (Gundry et al. 2007; Boyko et al. 2010; Vaysse et al. 2011), and, finally, whole genome sequencing (WGS) (Lindblad-Toh et al. 2005; Freedman et al. 2014; Plassais et al. 2019).

As a result of the extensive history of genetic studies in dogs, remarkable advances have been made toward the resolution of the canine phylogeny (vonHoldt et al. 2010; Parker et al. 2017) and the temporal, geographic, and demographic history of dog domestication (Freedman et al. 2014; Shannon et al. 2015; Skoglund et al. 2015). Studies suggest that dogs were initially domesticated from gray wolves 15,000 to 40,000 yr ago (Freedman et al. 2014; Skoglund et al. 2015; Freedman and Wayne 2017; Ostrander et al. 2019), with a rapid diversification of breeds occurring within the past few hundred years. Currently, about 400 dog breeds exist worldwide, 193 recognized by the American Kennel Club and 360 by the Fédération Cynologique Internationale. Breed classification schemes have been proposed based on occupation, morphology, and geographic origin (American Kennel Club 2007; Wucher et al. 2017). The most recent genetic analysis, encompassing nearly 200 breeds and populations, suggests a monophyletic origin for most modern breeds and provides data regarding their origins and timing (Parker et al. 2017). Clusters of genetically similar breeds were identified and assigned to clades, which often reflected occupational and geographical origins.

Targeted and genome-wide genotyping approaches have led to the discovery of nearly 400 variants associated with more than 270 traits, over 220 of which correspond to possible models for human diseases (Online Mendelian Inheritance in Animals [OMIA], Sydney School of Veterinary Science, https://omia.org/). Particularly, genome-wide association studies (GWASs) involving modest size cohorts of dogs have led to the identification of variants controlling a variety of morphological, behavioral, and disease traits (Akey et al. 2010; Vaysse et al. 2011; Rimbault et al. 2013; Hayward et al. 2016; MacLean et al. 2019; Plassais et al. 2019).

The recent and intense artificial selective pressure exerted on dogs has induced pronounced inter-breed phenotypic differences while preserving intra-breed homogeneity. This process makes dogs of the same breed more likely to share not only morphometric traits but also disease susceptibilities (Karlsson and Lindblad-Toh 2008; Chase et al. 2009; Akey et al. 2010; Boyko et al. 2010; Marchant et al. 2017; Mansour et al. 2018; Ostrander et al. 2019). The level of anatomic similarity among dogs of any one breed is sufficiently strong that genetic studies have been successfully executed using breed standards as phenotypes, thus unraveling the genetic bases of some complex traits such as body size or behavior (Akey et al. 2010; Boyko et al. 2010; Vaysse et al. 2011; Hayward et al. 2016; MacLean et al. 2019; Plassais et al. 2019), which remain elusive, even in humans.

However, all these analyses have been performed using a subset of indicative SNPs and, more recently, SNPs from WGSs (Jagannathan et al. 2019; Plassais et al. 2019), but other forms of genomic variation have rarely been studied systematically. In fact, there is still a lack of fine-scale, genome-wide analyses of any variants other than SNPs across dog breeds, a notable exception when compared to humans and other model organisms (Yalcin et al. 2011; Brown et al. 2012; Sudmant et al. 2015). Copy number variation (CNV) has been previously studied in canines to elucidate specific phenotypes (Karyadi et al. 2013; Arendt et al. 2014; Waldo and Diaz 2015; Deane-Coe et al. 2018). However, most studies have focused on the comparison of dogs and wolves using array-based technologies, rather than undertaking a comprehensive and unbiased examination of all CNVs across the genome of distinct breeds (Berglund et al. 2012; Schoenebeck et al. 2012). Most CNV-related studies published to date only aimed to identify segmentally duplicated regions and did not aim to produce quantitative copy-number (CN) genotypes (Quilez et al. 2012; Molin et al. 2014). Knowing the exact number of copies at a locus is crucial for an accurate comparison of closely related organisms, such as distinct dog breeds and wild canids.

Here, we present a fine-scale CNV map of over 300 canid samples using WGS to produce the most extensive, high-resolution CNV panel in dogs to date. We examine more than 145 individual breeds, as well as nonbreed dogs, including village dogs, dingoes, captive New Guinea singing dogs, and wild canids such as wolves. We employ this data set to determine the ability of CNVs to recreate a current dog phylogeny. Moreover, we test for breed-phenotype associations using an extensive data set of breed standards as individual phenotypes in the first CNV-based GWAS performed in dogs to date.

## Results

We created a fine-scale CNV map using a panel of 263 purebred dog genomes, 59 village dogs from diverse locations, and 17 gray and Tibetan wolves (Supplemental Fig. S1). All the samples were previously sequenced at moderate coverage (NCBI BioProject [https://www.ncbi.nlm.nih.gov/bioproject/] accession numbers: PRJNA232497, PRJNA448733, PRJNA186960, PRJNA176193, PRJNA192935, PRJNA233638, PRJNA247491, PRJNA263947, PRJNA261736, PRJEB6079, PRJEB6076, PRJEB2162, PRJNA188158, PRJNA208087, PRJEB5500) (Methods; Supplemental Data S1, S2; Kim et al. 2012; Zhang et al. 2014). We find over 95% concordance between the structural variants generated in this study and those that we previously reported (Serres-Armero et al. 2017). The breed frequencies of the main CNVs presented here have additionally been validated using array comparative genomic hybridization data (Supplemental Fig. S2; Nicholas et al. 2011; Berglund et al. 2012; Ramirez et al. 2014). All phenotypic analyses are based on breed standards from the American Kennel Club and the Fédération Cynologique Internationale, which were published previously (Methods; Supplemental Data S3; Plassais et al. 2019).

### Copy number statistics of modern dogs, village dogs, and wolves

We report a total of 348.26 Mb of CNVs larger than one kilobase pair across all samples, amounting to approximately 14.69% of the entire canine genome. Of note, the sex chromosomes and unassembled chromosomes have not been considered in this work. A total of 6765 events (169.96 Mb) with an average size of 25.53 kb are gains, and 66,254 events (126.94 Mb) with an average size of 2.36 kb are losses relative to the CanFam3.1 dog genome reference build (Table 1). Gains, defined as any region with CN above two, are more often shared across samples than losses, and therefore most rare variants (MAF < 0.05) tend to be deletions (Table 1). We note that the gains cluster together across most autosomes while losses do not (Fisher aggregated *P*-value_gains_ = 0.006 and *P*-value_losses_ = 0.906) (Supplemental Fig. S3), a phenomenon previously described in other species (Li et al. 2009; Upadhyay et al. 2017). In terms of possible function alterations, 4989 gains and 10,366 losses overlap with at least 5% of a gene annotation, although we note that this overlap most often involves introns. In contrast, 1362 gains overlap entire genes, as opposed to only 686 losses (Table 1), which suggests that the overlap between CN losses and entire genes could be constrained and possibly deleterious in dogs.

**Table 1.**
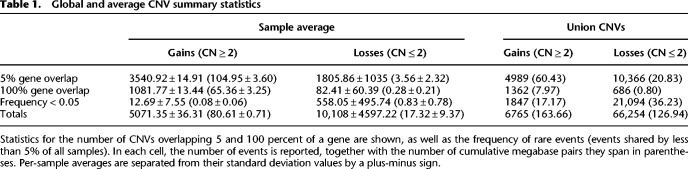
Global and average CNV summary statistics

We assessed how much of the current breed phylogeny, constructed using 150,000 SNPs (Parker et al. 2017), can be recapitulated using the duplications and deletions reported here (Supplemental Figs. S4, S5). Altogether, we are able to separate breeds resulting from the first domestication bottleneck (i.e., Arctic and Asian Spitz, Tibetan Mastiffs, and Ancient Sighthounds) (Freedman et al. 2014). However, we do not achieve a fully monophyletic separation of breeds derived in the eighteenth century and after, even when accounting for possible described admixture and inbreeding effects (Parker et al. 2017). Some possible explanations include homoplasies (Gazave et al. 2011; Bickhart et al. 2016), nonneutrality of the CNVs (Chen et al. 2009), and a poor genotype quality. Therefore, the correlation of CNV with geography and genealogy is not as clear as previously observed using single nucleotide variation (SNV) (Parker et al. 2017). This observation suggests that CNV variability is not strongly driven by population structure, as are SNVs. Consequently, population stratification is not expected to be a prevalent confounding factor in CN genotype-phenotype correlations within domestic breeds.

### Comparative analysis of modern dogs, village dogs, and wolves

**Figure 1.**
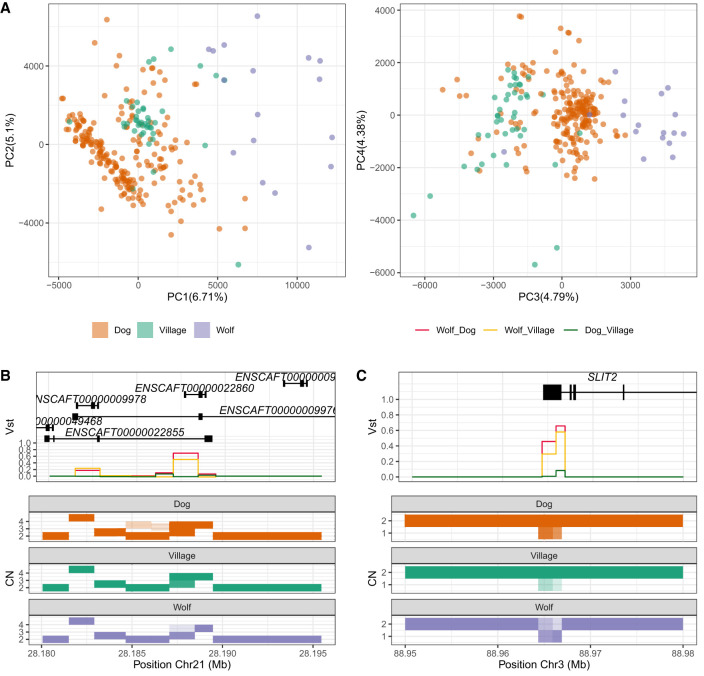
Global and specific differences in CN between breed dogs, village dogs, and wolves. (*A*) Copy number-based principal component analysis of breed dogs (orange), village dogs (green), and wolves (purple). (*B*,*C*) Depictions of the copy number values for two highly differentiated loci: the *HBB* chain gene cluster (ENSCAFT00000009978, ENSCAFT00000022860, ENSCAFT00000009984) and *SLIT2*. Discrete copy number values for all samples are depicted in rectangle plots. Purple: wolves, green: village dogs, orange: modern dogs. *Top* panel: V_ST_ values for the same genomic windows; *bottom* panel: copy number window values.

Domestic dogs, wolves, and, to a lesser extent, village dogs can be discriminated via principal component (PC) analysis or by pairwise Euclidean distance (Fig. 1A). Most domestic dogs cluster together across the first four PCs, with a few exceptions overlapping village dogs. Wolves exhibit a greater dispersion but still constitute a distinct group (Fig. 1A). The first principal component (PC1) recapitulates the variation cline expected to result from dog domestication, where nondomestic dogs appear between domestic dogs and wolves. In contrast, the third principal component (PC3) hints at the opposite pattern, which endorses our previous observation that dogs may preserve CNV similarities with wolves (Serres-Armero et al. 2017). We did not observe a significant reduction in the number of CNV sites in purebred dogs when compared to wolves (Supplemental Fig. S6). This is in stark contrast to the SNV decline reported using whole genome sequencing data in numerous domesticated organisms (Freedman et al. 2014; Makino et al. 2018). In fact, village dogs show a slightly reduced number of CNV sites compared to dogs and wolves.

We applied the pairwise V_ST_ statistic (Redon et al. 2006) to scan for regions overlapping genes with different CN genotypes between dogs and wolves. We identified 11 Mb of high V_ST_ CNVs overlapping with 61 genes. Some of these CNVs have previously been reported, such as those affecting the *AMY2B* (Chr 6: 46,948,529–46,957,042) or *MAGI2* (Chr 18: 18,447,653–18,449,673) genes (Chen et al. 2009; Arendt et al. 2014). However, we also discovered some novel gene-overlapping CNVs (Supplemental Table S1). We observed a large proportion of CNVs overlapping genes involved in fatty acid biosynthesis (GO *P*-value < 0.001), some of which have been previously reported (Axelsson et al. 2013). We also report differences in CNV within a hemoglobin chain gene cluster (Chr 21: 28,187,060–28,188,467) (ENSCAFT00000009978, ENSCAFT00000022860, ENSCAFT00000009984; CN below two in many domestic and village dogs) (Fig. 1B) and *SLIT2* (Chr 3: 88,965,914–88,966,914; CN below two in many wolves) (Fig. 1C) genes which, in humans, have been associated with adaptation to high altitude and neural development, respectively (Hu 1999; Bigham 2016). We note that the CN distribution for *SLIT2* in our panel is unexpected, because *Slit1/2* mice knockouts suffer optic chiasm and kidney development problems (Plump et al. 2002; Grieshammer et al. 2004), and a deletion segregating within the population at such frequencies would likely appear in homozygosity. A more cautious hypothesis would be to consider that this event corresponds to a sequence rearrangement or omission in the dog genome to which wolf reads map poorly.

### CNV-GWAS

Given the lack of global maps for genome-wide CNV analyses, absolute copy number has never been globally assessed for trait associations in dogs. Here, we used 58 nonredundant phenotypes based on breed standards in a search for associated CNVs. In order to assess different association trends, we implemented and compared discrete and continuous generalizations of widely used association tests (see Methods and Supplemental Table S3), controlling for population stratification only when an excess of significant *P*-values was observed after accounting for inflation (Tsepilov et al. 2013).

CNV associations were found for 31 of the 58 nonredundant phenotypes assayed. An additional 17 phenotypes showed associated CNVs with *P*-values above the secondary threshold (Methods), one order of magnitude below the Bonferroni correction. The most frequently represented phenotypes were body height, hair length, and tail-to-body ratio. In contrast, our analyses were not able to assess some commonly studied phenotypes such as eye pigmentation or fur color and density, for which phenotype-driving CNVs are known, for instance, those affecting the *ASIP*, *RALY*, *RSPO2, FOXI3,* and *ALX4* genes (Drögemüller et al. 2008; Cadieu et al. 2009; Dreger and Schmutz 2011; Deane-Coe et al. 2018). These traits are variable within breeds and thus require individual phenotypes in order to be resolved, or they are particular to a unique breed for which there were few representatives in our panel. Additionally, our median sample sequence coverage of 12.66× is unsuitable for accurately detecting CNVs below 500 bp. For these phenotypes, our panel and phenotype imputation approach using breed standards lacked the power to detect the associated CNVs.

**Figure 2.**
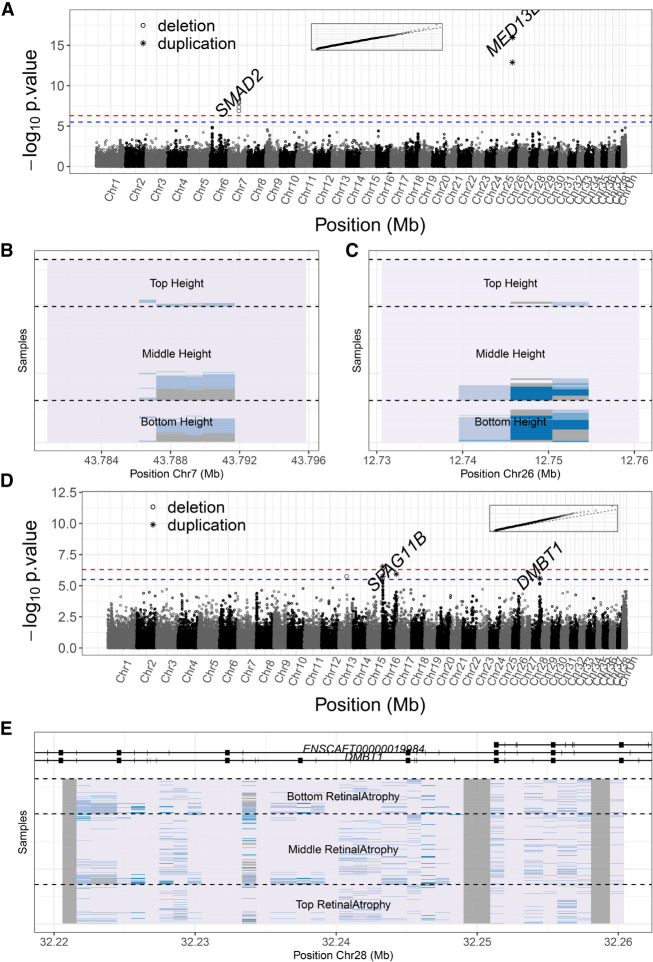
Analysis of the associations between CNVs and two phenotypes. (*A*,*D*) Manhattan plots of the copy number GWAS for breed standard height, retinal atrophy susceptibility, respectively (Jones et al. 2008). Red line: Bonferroni correction (−log_10_*P*-value = 6.417). Blue line: one order of magnitude below Bonferroni correction (−log_10_*P*-value = 5.417). *P*-values were calculated using different tests (Supplemental Table S3 and Methods). (*B*,*C*,*E*) Close-up of the relevant regions for each trait, respectively: *SMAD2* (Chr 7: 43,787,168–43,801,320) and *MED13L* (Chr 26: 12,739,546–12,754,676) loci for height, and *DMBT1* (Chr 28: 32,220,591–32,260,415) for retinal atrophy. Each sample corresponds to a line along the *y*-axis and is ordered according to the trait in question. The *x*-axis shows the genomic position of each window. CN windows for each sample are colored according to their normalized distance to the median CN in the window; the darker the shade of blue, the more the CN of a sample differs from the window median. Gray CN windows correspond to uncertain genotypes.

We report a duplication associated with body height (Chr 26: 12,739,546–12,754,676) (Fig. 2A,C; Supplemental Fig. S7A), previously reported as a SNP result (Hayward et al. 2016; Plassais et al. 2019), which harbors a CpG island 20 kb upstream of the *MED13L* gene. We detect a well-supported Hi-C interaction (Vietri Rudan et al. 2015) between this duplication and the Hi-C window containing the *TBX3* gene, located almost 1 Mb downstream (Supplemental Table S5). Haploinsufficiency of the syntenic region containing *TBX3* and *MED13L* (Jin et al. 2013) has been reported to cause short stature, developmental delay, and intellectual disability, among other conditions in humans (Adegbola et al. 2015), and is established as a major contributor to height in horses (Kader et al. 2016). This interaction contains a CCCTC-binding factor (CTCF) motif, which is conserved in placental mammals (Pollard et al. 2010), and contributes to the formation of a 3D chromatin regulatory domain that isolates *TBX3* from *MED13L* and *TBX5* in mouse (van Weerd et al. 2014).

About 50 small-sized dogs in our panel, defined as breeds with an average adult male and female height of 22 cm or less, carried either a homozygous or heterozygous ∼14-kb deletion (Chr 7: 43,787,168–43,801,320) located ∼10 kb downstream from the *SMAD2* gene. This deletion encompasses a CpG island on the 3′ end of the gene and has been previously hypothesized to have regulatory functions (Fig. 2A,B; Supplemental Fig. S7B; Rimbault et al. 2013). This region contains a conserved cluster of transcription binding sites located in a mammalian-level synteny block, and its corresponding orthologous region (mm10_Chr 18: 76,324,482–76,336,682) physically interacts with the promoter of the *HOXA10* gene in a different chromosome in mouse ESCs (Denholtz et al. 2013). *HOXA10* is a developmental gene essential for osteoblastogenesis and skeletal development (Favier et al. 1996; Hassan et al. 2007), as well as in sexual differentiation in mammals (Kobayashi and Behringer 2003; Wilhelm and Koopman 2006).

In this study, we sought to determine if genetic associations could be found for established breed propensities for cardiac, thyroid, orthopedic, and eye diseases (available from the Orthopedic Foundation for Animals [OFA]) using a similar approach as that described for morphological traits. A minimum threshold of one affected individual in 2000 (0.05%) reported in the OFA database was required for a breed to be included in the analysis of any disease. Also, we selected as “cases” breeds with the highest risk of developing the disorder, whereas breeds with the lowest risk served as “controls.” We report a minimum of 21 “case” breeds for any of the eight diseases analyzed here.

Consistent with the lack of clinical phenotypes in our study and the assumption that most dogs in the database were healthy, the GWAS performed here, especially those involving cardiac and thyroid conditions, suffered from a *P*-value deflation. However, our approach found a few noteworthy CNV candidates overlapping provocative candidate genes (Supplemental Table S2). We detected an association for generalized-progressive retinal atrophy (gPRA) risk in CNVs covering more than 10 exons of the *DMBT1* multicopy gene (Chr 28: 32,220,591–32,260,415) (Fig. 2D,E). Of note, the genetic basis of canine progressive retinal atrophy has for long been a field of intense study in humans and dogs (Acland et al. 1994; Lippmann et al. 2007; Downs et al. 2011; Bunel et al. 2019; Hitti et al. 2019). The lowest gPRA risk group (0.17% average prevalence), which included 34 breeds, had more copies of this complex duplication than the higher risk group (2.38% average prevalence), which included 26 breeds. This could suggest a potential protective role for an increased CN. CNVs in this gene have previously been hypothesized to be associated with macular degeneration in humans, a distinct condition but one which nevertheless affects the retina (Polley et al. 2016).

### CNV-GWAS annotation

To investigate the relationship between CNV- and SNP-driven associations, we gathered data on genomic variants from different studies to test whether our secondary-threshold GWAS associations followed any discernible patterns. In particular, we focused on signals that were close to significance, as concordance between multiple noncoding or intergenic regions and their previous, independent annotations could both serve as a validation and potentially point to polygenic effects.

We cross-referenced the CNV-GWAS signals with a preceding WGS-GWAS study for the same traits (Plassais et al. 2019). For each reported SNV association, we assessed whether the closest CNV signals had higher *P*-values than expected (Methods). Even if we were able to identify this trend in a few cases, most prominently deletions, the majority of CNV associations were independent of SNP associations (Supplemental Fig. S8). For instance, one of our most significant GWAS results, the *SMAD2* locus, segregates together with a previously reported SNP at frequencies of 0.6 ± 0.29 depending on the breed (Chase et al. 2009; Rimbault et al. 2013; Plassais et al. 2019).

In order to assess whether intergenic and intronic CNV associations could point to unannotated regulatory regions, we studied the enrichment in conserved motifs. For this, we intersected the 75-way GERP score Ensembl annotation (Hunt et al. 2018) with our significant calls (Methods). We found no significant increase in the number of conserved and associated CNVs compared to the global background of all nongenic structural variation (Supplemental Table S4). This means that associated CNVs behave the same as the rest of CN events in terms of sequence conservation. Indeed, there seems to be an overall depletion in highly conserved motifs in the canine structural variation space, in part due to the poor alignment within complex regions when constructing conservation scores. This depletion is consistent with previous findings involving ultraconserved elements in mammals (Derti et al. 2006), suggesting that dosage alteration of these elements via CNVs could have deleterious effects.

A substantial part of the CNV-GWAS associations reported here either overlapped or were close to long noncoding RNA (lncRNA) genes. Therefore, we assessed the concordance between the lncRNA tissue of expression and the CNV-GWAS trait as a possible indicator of a nonspurious distribution of these association results. We used the dog lncRNA database (Le Béguec et al. 2018) to annotate the GWAS significant signals within a 10-kb range of a lncRNA based on the tissue where the lncRNA is most abundant. We compared the empirical GWAS-lncRNA contingency table against an independent distribution of both features (Methods). We found that some trait associations were enriched in concordant lncRNA tissues (e.g., brain lncRNA expression for intelligence) while retaining the expected counts in all other tissues. Particular examples are adrenal gland expression for temperament associations and muscle, blood, and heart expression for racing, that is, whether a breed is commonly used for dog racing (Supplemental Fig. S9). Although these results should be interpreted cautiously due to the low robustness of sparse and low contingency table counts, the consistency between traits with no major protein-coding associations and lncRNA expression encourages further exploration.

**Figure 3.**
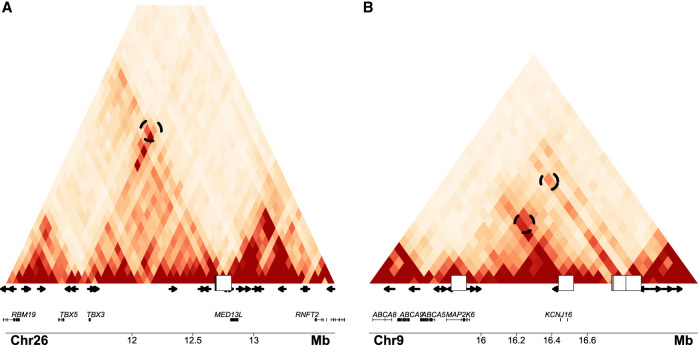
Instances of CNVs associated with phenotypes overlapping chromatin contacts. (*A*,*B*) Hi-C interaction heat maps for significant contacts on Chromosomes 26 and 9. The white squares on the *x*-axis mark the position the GWAS hits. The arrows mark the position and directionality of the most significant CTCF motifs of the area. The dashed circles within the Hi-C plot mark the significant interactions involving the relevant genes and the associated CNVs.

Finally, we also assessed whether any associated region, aside from *TBX3* mentioned above, displayed any distal Hi-C contacts (Fig. 3A). We verified all contacts reported here using ChIP-seq data for dog CTCF motifs (Schmidt et al. 2012), assessing whether each end of the contact contains at least one CTCF in inward opposing directions (Methods). Any long-range interactions involving lncRNAs were also accounted for in the corresponding analysis. We found seven significant, well-supported interactions in our data set. Most prominently, an association signal for hair length in a largely unannotated genomic region (Chr 9: 16,780,483–16,782,227) interacts with the *MAP2K6* gene located almost 1 Mb away (Fig. 3B). The role of the gene in hypertrichosis in both humans and foxes is a topic of debate (Clark et al. 2016). Of note, the *KCNJ2* and *KCNJ16* genes, together with four lncRNAs, are also within the range of this interaction.

### Breed V_ST_

We next analyzed possible differences in copy number arising from breed differentiation without consideration of any specific phenotype. We applied the pairwise V_ST_ statistic (Methods; Supplemental Data S4; Redon et al. 2006) to all pairs of breed clades consisting of more than six individuals (Methods). Overall, we found some highly differentiated loci in a subset of established clades (primarily Tibetan Mastiffs, Arctic Spitz, Shepherds, Ancient Sighthounds, and Scenthounds), largely corresponding to gene-poor regions. However, a few of these differentiated CNVs contained one or more members of extensive protein families (such as olfactory receptors, solute carriers, and late cornified envelope proteins).

**Figure 4.**
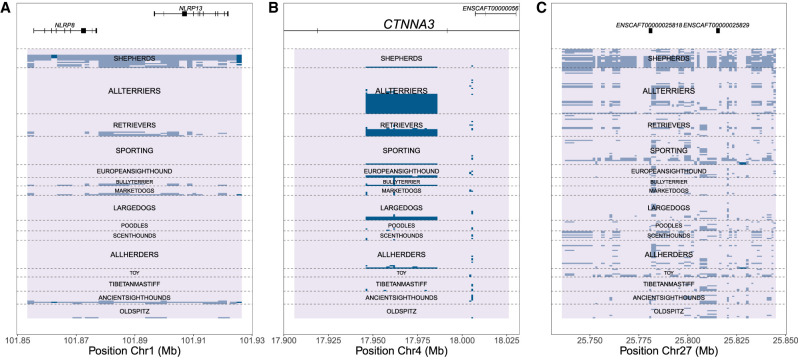
Instances of loci where specific clades have a different CN. (*A*–*C*) Representation of high inter-breed V_ST_ regions for the *NLRP8* and *NLRP13* genes, *CTNNA3*, and *SLC7A* (ENSCAFT00000025818 and ENSCAFT00000025829), respectively. Normalized copy number is represented by color, where the modal CN is the lightest color, and deviations from the mode (either deletions or duplications) are colored darker. Each row represents a sample ordered by clade, as described in Parker et al. (2017).

We detected a high V_ST_ signal in a CNV locus (Chr 1: 101,853,329–101,921,437) involving two adjoining genes involved in innate immune response, *NLRP13* and *NLRP8* (Fig. 4A). This association results from German Shepherds and Rottweilers having a different CN distribution than other breeds at this locus, probably due to their common ancestry. We also observed a deletion of ∼35 kb (Chr 4: 17,945,894–17,986,191) in the third intron of *CTNNA3* (Fig. 4B), a gene involved in cell adhesion. Terriers and retrievers possess, on average, fewer copies of this CNV than most other breeds. Both *CTNNA3* and *NLRP8/13* are secondary threshold GWAS associations for cataract propensity and herding (i.e., whether a breed is used for herding), respectively (Supplemental Table S2). Finally, we also found a homozygous deletion in many German Shepherds (Chr 27: 25,735,696–25,844,995) encompassing two *SLC7A* orthologs (ENSCAFT00000025818 and ENSCAFT00000025829) (Fig. 4C).

## Discussion

This study represents the first comprehensive and cohesive whole genome analysis specific to CNVs in the dog. We herein take advantage of our newly created map of structural variation in domestic dogs and other canids to explore the association between CNVs and phenotypes, showing clear and reproducible differences between established breed clades. GWASs with absolute CN as the testable variable are not common in the literature, especially outside of the field of human genetics (The Wellcome Trust Case Control Consortium 2010), and even less so in domesticated animals. In part, this is due to the technical difficulties of working with CNVs (Zhao et al. 2013) compared to SNPs and the more complex genetic scenario associated with their evolution (Sudmant et al. 2015; Xu et al. 2016). These hindrances ultimately result in a lack of specific tools and validation methods, and difficulties in the workflow. However, CN events have been correlated with distinct traits in a vast array of organisms (Hegele 2007; Karyadi et al. 2013; Chain et al. 2014; Upadhyay et al. 2017). Many structural variants and their phenotypic effects have been explored in dogs (Alvarez and Akey 2012), even shedding light on the molecular mechanisms of complex diseases such as skin cancer (Karyadi et al. 2013).

Using a curated panel of breed standards as a proxy for individual phenotypes, we discover new associations between phenotypes of interest and CNVs within or adjacent to excellent candidate genes. The replication of previous findings, especially those for achondroplasia, height, and body mass (Supplemental Fig. S7), provide proof of concept for this global approach. Our exploration of previously published chromatin contact maps for dogs and other mammals has provided additional insight into a distal association involving the candidate gene *MED13L* and has revealed candidate genes for hair length associations. Additionally, the CNV-GWAS for breed disease risks highlights some compelling associations, providing testable hypotheses in future studies.

Overall, we detect more than 110 clear instances (Supplemental Data S4) of CNVs that are at an increased frequency in a subset of modern dog clades. These CNVs are candidates for recent selection or could have been swept along in the process of clade origination. The much lower incidence of overall CNV events compared to other kinds of genomic variation, such as SNVs, suggests that CNVs may drive stronger phenotypic effects, and their evolution through domestication is a relevant topic in several organisms (Solé et al. 2019; Strillacci et al. 2019; Lye 2019). The inability to completely purge these potentially deleterious variants could result from a combination of artificial selective pressures and high inbreeding (Gaut et al. 2018), two phenomena that have been extensively reported in dogs (Calboli et al. 2008; Akey et al. 2010). Nevertheless, functional studies are needed to further validate such CNV candidates. Because of limitations in the number of dogs sequenced per breed, expanded data sets should be used to reexamine marginally significant results. In addition, realignment of the 341 sequences used here with long-read de novo assemblies (Wang et al. 2021) will further refine these results.

Finally, we highlight the importance of including multiomics data for conducting complete genetic analyses in any system, particularly those involving complex traits. The use of orthogonal genomic variants, such as tissue-specific lncRNA expression profiles, can help to contextualize the more abstruse CNV associations and explore their molecular bases. Whole genome copy number analyses provide a powerful approach for identifying the role of genomic variation in diversity within many understudied organisms.

## Methods

### Samples

The sequencing data used in this study can be downloaded from the NCBI BioProject database (https://www.ncbi.nlm.nih.gov/bioproject/) under accession numbers PRJNA232497, PRJNA448733, PRJNA186960, PRJNA176193, PRJNA192935, PRJNA233638, PRJNA247491, PRJNA263947, PRJNA261736, PRJEB6079, PRJEB6076, PRJEB2162, PRJNA188158, PRJNA208087, and PRJEB5500. The aCGH data used for validation (Supplemental Fig. S2) are available in the NCBI GEO DataSets database (https://www.ncbi.nlm.nih.gov/gds/) data set under accession numbers GSE26170, GSE40210, and GSE58195. The processed CNV files, the phenotype tables, the structural variants file, the differentiated CNV file, and the sample accessions file are available as Supplemental Data S1–S5. All accessions for the whole genome sequencing samples used in this study are detailed in the Methods section and Supplemental Data S5.

We analyzed a panel of 431 canid samples containing purebred dogs, free-ranging (village) dogs, and wolves. Four wolves (Wolf08, Wolf18, Wolf23, Wolf34) were used to train the Hidden Markov Model transition matrix (explained below) and discarded from the final panel. After quality control (described below), a total of 263 dog genomes, 59 village dogs, and 17 wolves were kept. The purebred dog samples classify into more than 130 breeds, which altogether can be divided into more than 30 breed clades (Supplemental Data S1). The breed status of each sample was used to infer its phenotype.

### Phenotypes

A database of anatomical, behavioral, and disease susceptibility records was composed for each dog breed for the association studies. For most morphometrics, we used a phenotype database containing information from the FCI (http://www.fci.be/en/Nomenclature/) and the AKC (https://www.akc.org/dog-breeds/) published by Plassais et al. (2019). Behavioral data were retrieved from the CBAR-Q survey (https://datarepository.wolframcloud.com/resources/C-BARQ-Survey). Temperament and intelligence data were available from the ATTS (http://atts.org/breed-statistics/) database and “The Intelligence of Dogs” book (Coren 1994), respectively. Disease susceptibility data were exclusively extracted from the OFA database (https://www.ofa.org/diseases/breed-statistics#detail). Data for purebred dog litter size were obtained from a comprehensive study on the litters from a variety of dog breeds (Borge et al. 2011). Additional morphometrics data from a previous publication (Jones et al. 2008) were included. Matching morphometrics data from multiple data sources were found to be consistent in most cases.

### Copy number genotyping

#### Sample preprocessing

The initial collection of sample sequencing formats was coerced into FASTQ format using the appropriate tools including biobambam (https://github.com/gt1/biobambam), qseq2fastq (https://github.com/ahcm/qseq2fastq), and fastq dump (https://github.com/ncbi/sra-tools), and all sequencing qualities were standardized to Phred 33 encoding. Adapters were trimmed with TrimGalore (Martin 2011), using paired-end data when possible and restricting the output length to a minimum of 36 base pairs. The trimmed sequencing reads were then further split into 78-mers to facilitate the mapping process.

#### Reference assembly preparation

In order to use an exhaustive mapper and further perform the necessary read depth calculations, the CanFam3.1 assembly was prepared as indicated below:

1. Standard repeat masking: masking of the corresponding genome-wide tandem repeat finder annotations (Haeussler et al. 2019).
2. Assembly *k*-mer masking: in order to identify potentially hidden repeats, the assembly was split into 36-mers with a 5-bp overlap and remapped against itself using GEM (Marco-Sola et al. 2012) at 6% divergence with a 10% edit distance. *k*-mers mapping to more than 20 positions were additionally masked. This version of the assembly was indexed using BWA (Li and Durbin 2009), GEM, and SAMtools (Takeuchi et al. 2016), and used for all subsequent sample mappings.
3. Padding and assembly windowing: all the masked locations described in steps one and two were extended for 78 bp on each side. This aims to correct for the general effect of read depth deflation around masked loci. Next, the assembly was partitioned into 1000-bp windows of nonmasked sequence as described in Alkan et al. (2009). The resulting 1-kb genomic window coordinates were used for copy number estimation and are theoretically comparable across samples due to the common reference.

#### Mapping and read depth postprocessing

The preprocessed samples were aligned against the masked CanFam3.1 reference using the GEM exhaustive mapper at 6% divergence and 10% edit distance. The resulting files were processed with mrCaNaVaR (Alkan et al. 2009), a tool for absolute copy number prediction based on read depth normalization, which performs GC correction and discriminates between CN 2 (a.k.a. control or diploid regions) and potentially duplicated windows.

#### Quality control

Three primary parameters were assessed to decide which samples to include in these analyses:

- CR deviations from a Gaussian distribution were measured using the Kolmogorov distance. Extreme deviations from a bell shape or distribution mean shifts could be a product of faulty normalization.
- A hard threshold (0.45) was imposed on the CR standard deviation to avoid excessive scatter of the HMM emissions.
- Local (i.e., neighboring window) control region copy number correlations were assessed using Pearson's coefficient. An excess of nonindependent and nonhomoscedastic CR windows was detected in a few samples, which were discarded.

#### Copy number genotyping and smoothing

We sought to discretize the copy number estimations to enhance comparability and produce a more biologically consistent CN measure. In order to do so, a similar setup to the one described in Serres-Armero et al. (2017) was used.

We implemented a Hidden Markov Model in which the observed read depth (emissions) was linked to a certain integer CN value (hidden state) via a Gaussian distribution. Briefly, a set of hidden states ranging from 0 to 20 (plus Gaussian mixtures of states with CN above 20) with variance proportional to the empirical diploid dispersion and the hidden CN was declared. The transition matrix was trained using the Baum-Welch algorithm coded in the Python pomegranate (https://github.com/jmschrei/pomegranate) package. Then, the forward-backward probability of each state for each 1-kb window was predicted in every sample.

Additionally, the CN genotypes were updated using the predicted probabilities of all samples together. A sliding window range of five windows with four window overlap was defined, and the expected joint probability of each state within it was computed. The expected local probabilities of each state were then used as priors to apply Bayes’ rule on the third window within the range for each sample. Finally, the range of CN states whose cumulative posterior distribution summed up to 0.95 was output.

$$p(CN=N|cn\in [x+dx])=\frac{\;p(cn\in [x+dx]|CN=N)p(CN=N)}{\;p(cn\in [x+dx])}=\frac{PDF\left(cn,N,\sqrt{0.5N}\sigma_{CR}\right)}{\sum_{CN}PDF\left(cn,N,\sqrt{0.5N}\sigma_{CR}\right)}p(N);$$

we defined as duplications any windows where at least one individual had a CN range above (and not overlapping) CN = 2. Similarly, all windows with a CN range below (and not overlapping) CN = 2 were considered deletions. Most analyses were restricted to the duplication/deletion space defined here.

#### Copy number classification and deletion recalling

Working with ranged CN genotypes can make it difficult to find natural sample clusters or perform genotype classification. Therefore, for each duplicated 1-kb window, the set of the most distant, nonoverlapping CN interval(s) compared to the modal CN was defined. The rest of the CN ranges were then assigned to any of the defined intervals based on the overlap, with the option to define intermediate, nonoverlapping intervals. The process was repeated until no range was reclassified.

Additionally, we aimed to emit definite, unranged genotypes for the set of deletions (defined via HMM) by refitting the empirical observations with a Gaussian Mixture Model. The R mixtools package was used (R Core Team 2018) to fit the mixture weights of a model with fixed means 0, 1, 2, and variances sd(CN = 2)/2, sd(CN = 2)/2, sd(CN = 2), where sd(CN = 2) is the standard deviation of the control region read depth. The expected probabilities of each CN averaged over all samples were used to update the individual probabilities on each site using Bayes’ rule, and only the most likely genotype was output.

### Segregation of structural variants by breeds

#### V_*ST*_ analyses

An in-house implementation of the pairwise V_ST_ statistic (Redon et al. 2006) was applied to each nondiploid 1-kb window in all breed clades containing six or more individuals. Much like *F*_ST_, V_ST_ compares the statistical variance of copy number values within each breed to that of both breeds taken together.

As we had previously detected that small sample sizes could bias the genomic V_ST_ distribution, all breed groups were subsampled to six individuals 1000 times, and the median V_ST_ value was kept for each window and comparison.

$$V_{ST}(B_{1},B_{2})=1-\frac{len(B_{1})Var(B_{1})+len(B_{2})Var(B_{2})}{len(\left[B_{1},B_{2}\right])Var(\left[B_{1},B_{2}\right])}.$$

#### PCA

Principal component analyses were performed using the prcomp function from the R stats package. In order to prevent sample size biases, a common PCA basis was created using a random balanced subset of all breed clades. All other samples were projected into this common basis by applying the centering, scaling, and rotation matrices output by prcomp.

### CNV-based phylogeny

#### Tree construction

All Euclidean distance matrices were calculated directly from the CN values using the R stats package. The distance matrices were then used to construct phylogenetic trees with the ape (Paradis et al. 2004) R package.

#### Tree comparisons

When trees containing different samples, breeds, and metrics had to be compared, we extracted the common tree topologies by projecting the different distance matrices against the column space of their respective indicator matrices (where each ordered column signals which samples belong to a common breed). The column values of the resulting matrices were collapsed by breed and propagated across the diagonal to create a symmetric, synthetic distance matrix which retains the topological properties of the original matrix. The resulting distance matrices were thus ordered, filtered, and comparable under common scaling conditions. In our case, we applied simple correlation and 2-norm comparisons.

$$B{\left(B^{T}B\right)}^{-1}B^{T}D,$$

$$\forall \mathrm{sample}\in \{1,2,\ldots I\},\forall \mathrm{breed}\in \{1,2,\ldots J\}b_{ij}:=\left\{\begin{array}{c} 1\;\mathrm{if}\;\mathrm{sample}\in \mathrm{breed}\\ 0\;\mathrm{if}\;\mathrm{sample}\notin \mathrm{breed} \end{array}\right\},$$

$$d_{ij}^{2}=CN_{sampi}-CN_{sampj}\begin{array}{c} 2\\ 2 \end{array}.$$

#### Haplotype sharing tree

In order to avoid the effect of possible excessive haplotype sharing across seemingly unrelated breeds on the tree topologies, these potentially confounding loci were omitted. For this, the positions of the pairwise shared haplotype locations in Parker et al. (2017) were removed from the deletion space, and the sample distances were recalculated based on the remaining deletions, correcting for the amount of subtracted positions. A similar setup was designed that removes haplotypes sharing breeds in any pairwise comparisons. The resulting topologies were compared as described above.

#### GWAS

Generalizations of three widely used statistical tests were used to accommodate population stratification into *P*-value calculations. The tests were chosen to match the requirements of each phenotype distribution, favoring linear regression for continuous, potentially additive traits, and χ^2^ independence tests for tabulated, categorical data. Stratification on categorical data was only applied if inflation was detected in the *P*-values (Supplemental Table S3).

A “primary” Bonferroni multiple-testing correction threshold (with value −log_10_ *P*-value = 6.417) was established by dividing the significance value (α = 0.05) by the total number of CNV windows. However, real CNV events are generally composed of many successive windows, and therefore, their *P*-values will be statistically nonindependent. As the total number of independent tests should be lower than the total number of windows, a “secondary” and more permissive threshold (with value −log_10_ *P*-value= 5.417) was also defined to be one order of magnitude below the primary. We considered *P*-values above either threshold to be of interest, prioritizing those above the primary threshold.

#### Categorical phenotypes

We applied an in-house implementation of the generalized Cochran–Mantel–Haenszel (CMH) test by Richard Landis (Landis et al. 1979), as explained in Alan Agresti's 2002 statistical handbook (Agresti 2002). This generalization allows for stratification of data into subpopulations and with the ordinal nature of phenotypes and copy numbers.

The phenotype data were split into the top 70 and bottom 30 percentiles (two groups). Copy number was also classified into categories, as previously described. When necessary, population stratification was accounted for by dividing the data into two similarly sized substrata based on the breed tree proposed by Parker et al. (2017).

#### Continuous phenotypes

Assessment of specific copy number and phenotype trends was carried out using linear regression (R base software). The four first principal components of the scaled copy number data matrix (see below) were used as covariates to correct for population stratification. Regression analyses and any further GWAS recalculations were restricted to the duplications/deletions space.

### GWAS comparisons and validations

All genome arithmetics were performed using the BEDTools suite (Quinlan and Hall 2010), enforcing the necessary parameters. In broad terms, window-based association signals were mapped to their respective structural variants and then intersected with the corresponding annotation files.

#### lncRNA

We proposed the independent joint distribution of all copy number lncRNA tissues and the proportion of association signals across traits as the null hypothesis to test for deviations in the associated copy number variant (ptissue ⊗ passociation). Multinomial distributions over the association table were assumed, and excessive cell counts were reported in terms of standard deviations. lncRNA data were downloaded from Le Béguec et al. (2018).

#### Conservation scores

Highly conserved regions were defined by binning GERP scores according to their 95th quantile value (∼3). All nonexonic structural variants were used as a background to test whether nonexonic associated variants were enriched in highly conserved elements. We tested the null hypothesis of variable independence using Fisher's test (variables: association and conservation). GERP scores were downloaded from Hunt et al. (2018).

#### Hi-C

The ratio of main contact read support (region against itself) and every other region involving that same contact was computed in CNV regions. A threshold was set at the 95th quantile of the distribution to call significant contacts (Supplemental Fig. S10). Hi-C data were downloaded from Vietri Rudan et al. (2015).

#### ChIP-seq

All putative significant contacts were verified by assessing both that they contained at least one CTCF motif on each side (Vietri Rudan et al. 2015) and that the CTCF motifs were correctly oriented, that is, facing each other. The ChIP-seq data were downloaded from Schmidt et al. (2012) and lifted over from the CanFam2 genome build to CanFam3.1 (Haeussler et al. 2019). We reannotated the CTCF orientation for the relevant loci using the dog-specific CTCF position weight matrix (https://www.ebi.ac.uk/research/flicek/publications/FOG03) and the software PWMTools (Ambrosini 2018).

#### Leading SNP

We gathered all structural variation GWAS *P*-values within 1 Mb surrounding the leading SNP GWAS signals proposed by Plassais et al. (2019). Next, for each leading SNP, the structural variation data were binned into equally sized blocks to assess if the block containing the leading SNP contained more significant *P*-values than the rest.

## Supplementary Material

Supplemental Material
