# Supplementary material for: Copy number variation underlies complex phenotypes in domestic dog breeds and other canids
Source: Genome Res. 2021 May;31(5):762–74. doi: 10.1101/gr.266049.120 (PMC8092016; doi:10.1101/gr.266049.120)
Supplement: Supplemental Material [file supp_31_5_762__index.html]

Copy number variation underlies complex phenotypes in domestic dog breeds and other canids — Supplemental Material 

# Copy number variation underlies complex phenotypes in domestic dog breeds and other canids

## Supplemental Material

- Supplemental\_Data.zip
- Supplemental\_Material.pdf
