# Supplementary material for: Copy number variation underlies complex phenotypes in domestic dog breeds and other canids
Source: Genome Res. 2021 May;31(5):762–74. doi: 10.1101/gr.266049.120 (PMC8092016; doi:10.1101/gr.266049.120)
Supplement: Supplemental Material [file supp_gr.266049.120_Supplemental_Material.pdf]

Supplemental Material

Dog breed variation in genomic copy number underlies complex and novel phenotype associations

Aitor Serres-Armero[1], David Juan[1], Brian W. Davis[2,3], Inna S. Povolotskaya[4], Carlos Morcillo-Suarez[1], Jocelyn Plassais[2], Elaine A. Ostrander[2§] and Tomas Marques-Bonet[1,5,6,7§]

[1] IBE, Institut de Biologia Evolutiva (Universitat Pompeu Fabra/CSIC), Ciencies Experimentals i de la Salut, Barcelona, 08003, Spain

[2] Cancer Genetics and Comparative Genomics Branch, National Human Genome Research Institute, National Institutes of Health, Bethesda, MD, 20892, USA

[3] Department of Veterinary Integrative Biosciences, College of Veterinary Medicine, Texas A&M University, College Station, TX 77843

[4] Veltischev Research and Clinical Institute for Pediatrics of the Pirogov Russian National Research Medical University, Moscow, Russia

[5] CNAG-CRG, Centre for Genomic Regulation (CRG), The Barcelona Institute of Science and Technology (BIST), Barcelona, Spain

[6] Institucio Catalana de Recerca i Estudis Avançats (ICREA), Barcelona, Catalonia 08010, Spain

[7] Institut Català de Paleontologia Miquel Crusafont, Universitat Autònoma de Barcelona, Cerdanyola del Vallès, Catalonia, Spain

[§] Corresponding authors

Email addresses:

ASA: aitor.serres@upf.edu

BWD: bdavis@cvm.tamu.edu

ISP: ipovolotskaya@gmail.com

CMS: carlos.morcillo@upf.edu

JP: jocelyn.plassais@gmail.com

DJ: david.juan@upf.edu

EAO: eostrand@mail.nih.gov

TMB: tomas.marques@upf.edu

Table of contents

Supplemental data:

Supplemental Data S1 – Phenotype table for all samples and traits

Supplemental Data S2 – Copy number calls for all individuals

Supplemental Data S3 – Segmental duplication track for the study

Supplemental Data S4 – Top 0.1% differentiated windows across breed macrogroups containing more than 9 samples

Supplemental Data S5 – Availability and origin of the samples used for the study

Supplemental Tables:

Supplemental Table 1 – Top 0.1 % differentiated windows across dogs, village dogs and wolves

Supplemental Table 2 – GWAS results table overlapping functional element annotations

Supplemental Table 3 – GWAS phenotypes and tests

Supplemental Table 4 – Contingency table of associated CN and highly conserved regions

Supplemental Table 5 – Association signals within significant Hi-C contacts

Supplemental Figures:

Supplemental Figure 1 – Number of samples and phenotypes for analysis

Supplemental Figure 2 – CNV validation

Supplemental Figure 3 – CNV distance by chromosome

Supplemental Figure 4 – CNV based breed phylogeny

Supplemental Figure 5 – Correlations between CNV and SNV phylogenies

Supplemental Figure 6 – Proportion of CNV sites in dogs, village dogs and wolves

Supplemental Figure 7 – CNV interactions between standard breed height genes

Supplemental Figure 8 – P-value of associated CNV relative to leading SNV distance

Supplemental Figure 9 – Correlations between lncRNA experssion and CNV associations

Supplemental Figure 10 – Distribution of CNV Hi-C contacts relative to main contact

Chr	Start	End	Wolf_Dog	mean(Wolf)	mean(Dog)	Wolf_Village	mean(Wolf)	mean(Village)	Dog_Village	mean(Dog)	mean(Village)	EnsemblID	protein/description
Chr1	7322992	7324705	0.663	0.931	1.681	0.427	0.931	1.591	-0.014	1.681	1.591	NA	NA
Chr1	36458286	36459286	0.124	5.597	5.199	0.111	5.597	6.137	0.317	5.199	6.137	NA	NA
Chr1	53756693	53757943	0.613	1.175	1.815	0.594	1.175	1.679	0.049	1.815	1.679	NA	NA
Chr1	64439302	64441515	0.674	1.086	1.806	0.435	1.086	1.676	0.003	1.806	1.676	ENSCAFT00000057044	NA
Chr1	67106245	67109181	0.655	0.991	1.775	0.464	0.991	1.649	-0.001	1.775	1.649	CpG:19,CpG:29	NA
Chr1	70810119	70812745	0.657	1.152	1.861	0.367	1.152	1.684	0.018	1.861	1.684	NA	NA
Chr1	97707018	97708444	0.053	3.225	3.691	0.578	3.225	4.692	0.308	3.691	4.692	NA	NA
Chr1	104116423	104117423	-0.015	11.922	11.562	0.275	11.922	13.588	0.314	11.562	13.588	NA	NA
Chr1	104168177	104169949	-0.019	12.864	12.468	0.303	12.864	15.271	0.308	12.468	15.271	NA	NA
Chr1	104403461	104405234	-0.019	12.852	12.462	0.290	12.852	15.315	0.301	12.462	15.315	NA	NA
Chr1	105158895	105160977	-0.019	2.282	2.230	0.307	2.282	3.048	0.318	2.230	3.048	NA	*ZNF614*(zinc finger protein 615)
Chr1	109620332	109622064	0.710	0.993	1.658	0.633	0.993	1.647	-0.019	1.658	1.647	CpG:147,*ENSCAFT00000006986*,*ENSCAFT00000042940*,*ENSCAFT00000045979*	CCDC61(coiled-coil domain containing 61),PGLYRP1(peptidoglycan recognition
Chr1	117012318	117013318	-0.013	2.527	2.622	0.475	2.527	3.219	0.334	2.622	3.219	NA	NA
Chr1	117038815	117039815	-0.021	2.497	2.510	0.474	2.497	3.166	0.381	2.510	3.166	NA	NA
Chr1	119420548	119425739	0.660	1.212	1.906	0.338	1.212	1.726	0.036	1.906	1.726	CpG:22	NA
Chr2	46279374	46281321	0.719	1.091	1.910	0.455	1.091	1.822	-0.016	1.910	1.822	NA	NA
Chr2	84006412	84007412	0.014	3.085	3.616	0.339	3.085	1.941	0.294	3.616	1.941	*ENSCAFT00000025995*,*ENSCAFT00000026009*,CpG:25	*VPS13D*(vacuolar protein sorting 13 homolog D)
Chr2	84006412	84007412	0.014	3.085	3.616	0.339	3.085	1.941	0.294	3.616	1.941	*ENSCAFT00000025995*,*ENSCAFT00000026009*,CpG:25	*VPS13D*(vacuolar protein sorting 13 homolog D)
Chr3	17605975	17609161	0.703	1.249	2.117	0.441	1.249	1.969	0.003	2.117	1.969	NA	NA
Chr3	27967323	27972116	0.654	1.366	1.999	0.383	1.366	1.802	0.066	1.999	1.802	NA	NA
Chr3	38552512	38555679	0.663	1.145	1.860	0.472	1.145	1.679	0.039	1.860	1.679	NA	NA
Chr3	88965914	88966914	0.658	1.072	1.798	0.580	1.072	1.636	0.082	1.798	1.636	*ENSCAFT00000026343*,CpG:405,*ENSCAFT00000044488*	*SLIT2*(slit guidance ligand 2)
Chr4	34894690	34895876	0.657	1.128	1.852	0.441	1.128	1.586	0.129	1.852	1.586	ENSCAFT00000036969	NA
Chr4	82979182	82981060	0.606	1.249	1.826	0.588	1.249	1.894	-0.012	1.826	1.894	NA	NA
Chr5	82485750	82487727	0.514	1.355	2.156	0.090	1.355	1.604	0.277	2.156	1.604	*ENSCAFT00000032498*,CpG:91,*ENSCAFT00000032493*	*TERB1*(telomere repeat binding bouquet formation protein 1),NAE1(NEDD8 acti
Chr6	11325095	11329538	0.698	1.174	1.906	0.487	1.174	1.726	0.056	1.906	1.726	NA	NA
Chr6	14962741	14964823	-0.003	28.466	27.968	0.260	28.466	30.474	0.302	27.968	30.474	NA	NA
Chr6	46948529	46957042	0.716	2.818	12.433	0.679	2.818	11.785	-0.015	12.433	11.785	ENSCAFT00000044827	*AMY2B*-like(pancreatic alpha-amylase)
Chr7	24874718	24879432	0.690	2.174	4.036	0.178	2.174	2.931	0.236	4.036	2.931	NA	NA
Chr7	47404240	47406439	0.672	1.019	1.831	0.507	1.019	1.817	-0.020	1.831	1.817	NA	NA
Chr8	16078659	16081080	0.619	1.380	2.376	0.641	1.380	2.187	0.020	2.376	2.187	*ENSCAFT00000021805*,CpG:298	*FOXA1*(forkhead box A1)
Chr8	30090421	30094950	0.091	33.545	31.850	0.161	33.545	35.949	0.351	31.850	35.949	NA	NA
Chr8	36829253	36830901	0.621	1.074	1.745	0.584	1.074	1.746	-0.019	1.745	1.746	NA	NA
Chr8	71993201	71994594	0.605	1.051	1.671	0.647	1.051	1.732	-0.009	1.671	1.732	CpG:21	NA
Chr9	7515709	7521860	0.675	12.171	5.417	0.725	12.171	4.776	0.002	5.417	4.776	NA	NA
Chr9	7807191	7808191	0.478	7.030	3.231	0.604	7.030	2.556	0.014	3.231	2.556	NA	NA
Chr9	7909853	7922076	0.613	12.037	5.553	0.710	12.037	4.713	0.010	5.553	4.713	ENSCAFT00000058411	lincRNA
Chr9	7921076	7922076	0.587	9.027	4.178	0.704	9.027	3.368	0.024	4.178	3.368	NA	NA
Chr9	8719812	8720812	0.528	10.410	4.264	0.641	10.410	3.413	0.017	4.264	3.413	NA	NA
Chr9	8788677	8814741	0.671	14.779	6.217	0.727	14.779	5.368	0.008	6.217	5.368	NA	NA
Chr9	16653738	16680838	0.670	14.936	6.268	0.725	14.936	5.428	0.007	6.268	5.428	NA	NA
Chr9	17477075	17478075	0.604	9.086	4.150	0.717	9.086	3.339	0.024	4.150	3.339	NA	NA
Chr9	17477075	17488987	0.623	11.769	5.393	0.715	11.769	4.585	0.009	5.393	4.585	NA	NA
Chr9	17745656	17746656	0.689	6.901	3.268	0.686	6.901	3.330	-0.019	3.268	3.330	NA	NA
Chr9	34396314	34398643	0.678	1.170	1.816	0.474	1.170	1.806	-0.019	1.816	1.806	ENSCAFT00000028050	RPS6KB1(ribosomal protein S6 kinase B1)
Chr10	354874	356495	0.690	1.394	1.960	0.542	1.394	2.049	-0.005	1.960	2.049	ENSCAFT00000046809	IKZF4(IKAROS family zinc finger 4)
Chr10	13382577	13389042	0.644	1.159	1.733	0.662	1.159	1.836	0.010	1.733	1.836	ENSCAFT00000000735,ENSCAFT00000043266	TBC1D15(TBC1 domain family member 15)
Chr10	39045211	39050432	0.691	1.964	2.950	0.422	1.964	2.752	0.013	2.950	2.752	NA	NA
Chr10	53075791	53076791	0.000	4.707	4.874	0.504	4.707	5.579	0.315	4.874	5.579	NA	NA
Chr11	30641431	30643402	0.392	1.169	1.655	0.676	1.169	2.015	0.178	1.655	2.015	NA	NA
Chr11	32595538	32596538	0.032	17.825	17.176	0.226	17.825	19.255	0.335	17.176	19.255	NA	NA
Chr11	35451578	35453023	0.673	1.126	1.795	0.453	1.126	1.690	-0.001	1.795	1.690	NA	NA
Chr11	50164635	50167409	0.080	15.735	15.082	0.198	15.735	16.719	0.355	15.082	16.719	ENSCAFT00000002868	DNAJA1(DnaJ heat shock protein family (Hsp40) member A1)
Chr11	73614848	73616248	0.645	1.096	1.890	0.630	1.096	1.774	0.011	1.890	1.774	CpG:150,ENSCAFT00000005724	CDK5RAP2(CDK5 regulatory subunit associated protein 2)
Chr12	2499893	2502146	0.336	1.346	1.776	0.600	1.346	2.064	0.153	1.776	2.064	ENSCAFT00000043628	DLA-DMB(major histocompatibility complex, class II, DM beta)
Chr12	28398513	28400330	0.655	1.165	1.845	0.536	1.165	1.776	-0.010	1.845	1.776	NA	NA
Chr12	66553551	66555031	0.690	1.019	1.691	0.490	1.019	1.602	-0.009	1.691	1.602	ENSCAFT00000045616,ENSCAFT00000006183	FIG4(FIG4 phosphoinositide 5-phosphatase)
Chr13	13428884	13431336	0.145	25.072	23.784	0.076	25.072	26.025	0.329	23.784	26.025	ENSCAFT00000004038	DNAJA1-like(DnaJ heat shock protein family (Hsp40) member A1)
Chr13	18784265	18787101	0.673	1.418	2.141	0.637	1.418	2.165	-0.018	2.141	2.165	ENSCAFT00000001363	TAF2(TATA-box binding protein associated factor 2)
Chr13	44381011	44382618	0.668	0.902	1.627	0.434	0.902	1.464	0.019	1.627	1.464	ENSCAFT00000003138,ENSCAFT00000003145	FRYL(FRY like transcription coactivator)
Chr13	54108766	54110955	0.582	1.163	1.902	0.584	1.163	2.029	-0.004	1.902	2.029	NA	NA
Chr14	10357104	10363387	0.655	1.172	1.979	0.417	1.172	1.770	0.038	1.979	1.770	NA	NA
Chr14	15093068	15095238	0.477	1.315	1.792	0.598	1.315	2.008	0.057	1.792	2.008	NA	NA
Chr14	45898456	45900553	0.655	1.470	2.106	0.432	1.470	2.011	-0.006	2.106	2.011	NA	NA
Chr14	59190495	59191687	0.729	0.851	1.634	0.549	0.851	1.522	0.000	1.634	1.522	NA	NA
Chr15	16995561	16998768	0.706	2.305	3.086	0.520	2.305	3.012	-0.013	3.086	3.012	CpG:85,ENSCAFT00000008596	family with sequence similarity 183 member A
Chr15	58368063	58370151	0.692	1.184	2.081	0.560	1.184	2.075	-0.021	2.081	2.075	NA	NA
Chr16	3799547	3802414	0.711	1.218	1.862	0.439	1.218	1.723	0.026	1.862	1.723	NA	NA
Chr16	23714721	23716331	0.677	0.979	1.659	0.473	0.979	1.504	0.032	1.659	1.504	ENSCAFT00000009115	KAT6A(lysine acetyltransferase 6A)
Chr16	55234230	55239161	0.693	1.271	2.038	0.554	1.271	1.841	0.057	2.038	1.841	NA	NA
Chr16	57082651	57085376	0.669	1.251	1.934	0.589	1.251	1.858	-0.010	1.934	1.858	NA	NA
Chr17	163424	165290	0.651	1.286	2.035	0.600	1.286	2.005	-0.019	2.035	2.005	CpG:27	NA
Chr17	1421982	1426506	0.685	1.359	2.228	0.405	1.359	1.970	0.061	2.228	1.970	NA	NA
Chr17	1838558	1840163	0.665	1.230	1.890	0.572	1.230	1.733	0.062	1.890	1.733	CpG:242	NA
Chr17	33819130	33825066	0.663	1.467	2.379	0.339	1.467	2.082	0.065	2.379	2.082	NA	NA
Chr17	35161036	35162036	0.310	1.348	0.884	0.629	1.348	0.639	0.131	0.884	0.639	NA	NA
Chr17	52833733	52834912	0.181	5.869	5.391	0.062	5.869	6.255	0.303	5.391	6.255	NA	NA
Chr18	18474870	18476833	0.441	4.929	3.024	0.728	4.929	2.301	0.130	3.024	2.301	NA	NA
Chr18	18562040	18563040	0.580	14.872	6.449	0.700	14.872	4.774	0.086	6.449	4.774	NA	NA
Chr18	18697105	18698105	0.470	6.690	2.895	0.596	6.690	2.193	0.025	2.895	2.193	NA	NA
Chr18	26058943	26061619	0.656	0.800	1.509	0.394	0.800	1.359	0.007	1.509	1.359	ENSCAFT00000010796,OREG1321364,CpG:113	HSD17B12(hydroxysteroid 17-beta dehydrogenase 12)
Chr18	40756347	40759166	0.421	7.807	5.160	-0.016	7.807	7.521	0.325	5.160	7.521	ENSCAFT00000044337	olfactory receptor 4C11-like
Chr18	40775977	40779811	0.372	8.364	5.992	-0.010	8.364	8.265	0.316	5.992	8.265	ENSCAFT00000045137	olfactory receptor 4C11-like
Chr18	40837604	40840723	0.380	7.998	5.756	-0.024	7.998	7.880	0.305	5.756	7.880	ENSCAFT00000048359	olfactory receptor 4C11-like
Chr18	40992396	40993626	0.245	10.273	8.945	0.001	10.273	10.691	0.317	8.945	10.691	NA	NA
Chr18	45957644	45958644	0.587	1.479	2.395	0.590	1.479	2.194	0.036	2.395	2.194	CpG:108	NA
Chr18	52498390	52501710	0.506	1.261	1.796	0.583	1.261	1.974	0.031	1.796	1.974	NA	NA
Chr19	3791689	3793387	0.777	0.945	1.719	0.591	0.945	1.652	-0.014	1.719	1.652	NA	NA
Chr20	32237476	32239303	0.651	0.962	1.714	0.631	0.962	1.692	-0.018	1.714	1.692	NA	NA
Chr20	49564683	49568016	0.716	3.572	5.065	0.550	3.572	4.616	0.131	5.065	4.616	CpG:55	NA
Chr20	49624320	49629455	0.661	1.071	1.776	0.200	1.071	1.424	0.168	1.776	1.424	ENSCAFT00000048964	NA
Chr21	5962667	5964852	0.595	1.221	1.864	0.596	1.221	1.837	-0.018	1.864	1.837	CpG:65	NA
Chr21	28187060	28188467	0.694	2.191	3.308	0.502	2.191	3.245	-0.017	3.308	3.245	ENSCAFT00000022860	HBB1-like(hemoglobin subunit beta-like)
Chr22	21795805	21798085	0.678	1.376	1.976	0.640	1.376	2.061	-0.010	1.976	2.061	NA	NA
Chr23	24025972	24030579	0.700	2.246	3.128	0.455	2.246	2.989	-0.002	3.128	2.989	NA	NA
Chr23	41241407	41244502	0.437	2.211	1.546	0.603	2.211	1.325	0.024	1.546	1.325	NA	NA
Chr23	42533668	42536816	0.662	0.554	1.371	0.483	0.554	1.255	-0.003	1.371	1.255	CpG:76,ENSCAFT00000042806,CpG:109	NA
Chr24	8294149	8305224	0.093	2.117	1.673	0.597	2.117	0.792	0.220	1.673	0.792	NA	NA
Chr24	20586293	20589547	0.684	1.362	2.101	0.489	1.362	1.978	0.003	2.101	1.978	NA	NA
Chr25	89316	98110	0.204	28.066	26.231	0.067	28.066	29.190	0.365	26.231	29.190	NA	NA
Chr25	39687146	39689855	0.611	1.021	1.731	0.592	1.021	1.637	0.006	1.731	1.637	ENSCAFT00000016522,CpG:334	IRS1(insulin receptor substrate 1)
Chr25	43002746	43006942	0.681	1.342	2.081	0.496	1.342	1.992	-0.009	2.081	1.992	ENSCAFT00000044920,CpG:80	PSMD1(proteasome 26S subunit, non-ATPase 1)
Chr25	47325980	47327925	0.711	0.708	1.742	0.508	0.708	1.475	0.072	1.742	1.475	CpG:51,CpG:53	NA
Chr25	49327762	49328954	0.627	1.303	1.936	0.599	1.303	1.871	-0.006	1.936	1.871	CpG:6582	NA
Chr26	25788325	25794696	0.173	12.655	7.731	0.017	12.655	10.396	0.313	7.731	10.396	NA	NA
Chr26	25914450	25915450	0.142	11.475	7.489	-0.004	11.475	10.002	0.323	7.489	10.002	NA	NA
Chr26	26289201	26291297	0.133	11.772	8.066	0.002	11.772	10.219	0.292	8.066	10.219	NA	NA
Chr26	37982978	37985014	0.669	1.032	1.736	0.386	1.032	1.575	0.024	1.736	1.575	CpG:92	NA
Chr26	38566738	38569565	0.659	1.179	2.032	0.321	1.179	1.882	-0.007	2.032	1.882	NA	NA
Chr27	335782	338194	0.726	1.335	2.061	0.559	1.335	2.094	-0.019	2.061	2.094	NA	NA
Chr27	4648519	4649912	0.633	0.779	1.331	0.584	0.779	1.465	0.014	1.331	1.465	NA	NA
Chr27	18150820	18152905	0.684	1.338	2.133	0.403	1.338	1.881	0.075	2.133	1.881	NA	NA
Chr27	18668722	18670408	0.675	1.054	1.888	0.358	1.054	1.653	0.044	1.888	1.653	ENSCAFT00000017392	FAR2(fatty acyl-CoA reductase 2)
Chr28	38547990	38549193	0.670	0.818	1.533	0.423	0.818	1.267	0.129	1.533	1.267	CpG:66	NA
Chr29	7184973	7186415	0.650	1.064	2.239	0.727	1.064	2.187	-0.018	2.239	2.187	ENSCAFT00000011215,CpG:22	RAB28(RAB28, member RAS oncogene family)
Chr29	7184973	7191871	0.617	1.457	2.518	0.713	1.457	2.555	-0.013	2.518	2.555	ENSCAFT00000011215,ENSCAFT00000011219,CpG:22	RAB28(RAB28, member RAS oncogene family),TMEM68(transmembrane prote
Chr29	9164710	9167179	0.655	0.949	1.610	0.531	0.949	1.621	-0.020	1.610	1.621	OREG1316046	NA
Chr30	1484324	1486165	0.693	1.055	1.751	0.450	1.055	1.581	0.034	1.751	1.581	NA	NA
Chr30	40123523	40129151	0.388	1.644	2.310	-0.021	1.644	1.691	0.307	2.310	1.691	ENSCAFT00000028835,CpG:223	FBXO22(F-box protein 22)
Chr31	11015408	11017150	0.596	1.094	1.731	0.588	1.094	1.735	-0.018	1.731	1.735	NA	NA
Chr32	648942	649942	0.023	35.362	34.298	0.219	35.362	37.760	0.356	34.298	37.760	NA	NA
Chr33	2061220	2062661	0.653	1.129	1.829	0.477	1.129	1.748	-0.010	1.829	1.748	NA	NA
Chr33	10045590	10047669	0.003	18.408	17.961	0.269	18.408	20.046	0.319	17.961	20.046	NA	NA
Chr34	10810303	10811918	0.681	1.244	2.016	0.525	1.244	1.859	0.026	2.016	1.859	NA	NA
Chr35	24921155	24922747	0.602	1.135	1.768	0.603	1.135	1.689	-0.004	1.768	1.689	CpG:62,ENSCAFT00000042367	miRNA
Chr36	15258328	15259328	0.655	1.005	2.045	0.492	1.005	1.670	0.146	2.045	1.670	CpG:480,ENSCAFT00000019915	SP5(Sp5 transcription factor)
Chr36	22907674	22911117	0.679	1.324	2.054	0.580	1.324	1.913	0.025	2.054	1.913	CpG:227	NA
Chr38	23527587	23529271	0.659	1.036	1.799	0.512	1.036	1.562	0.122	1.799	1.562	CpG:6590,ENSCAFT00000018111	KIRREL1(kirre like nephrin family adhesion molecule 1)
ChrUn_AAEX03020568	0	37294	0.716	2.817	12.648	0.669	2.817	11.618	-0.005	12.648	11.618	ENSCAFT00000044657,ENSCAFT00000047259,ENSCAFT00000046326	pancreatic alpha-amylase
ChrUn_AAEX03022739	3277	16964	0.716	2.669	11.343	0.667	2.669	10.409	-0.003	11.343	10.409	ENSCAFT00000036352	pancreatic alpha-amylase
ChrUn_AAEX03023883	7210	10043	0.410	8.193	5.579	-0.018	8.193	7.979	0.323	5.579	7.979	ENSCAFT00000046868	olfactory receptor 4C11-like
ChrUn_AAEX03024353	0	8398	0.715	2.775	12.629	0.665	2.775	11.490	-0.002	12.629	11.490	ENSCAFT00000039013	pancreatic alpha-amylase

Supplemental Table S1 – Table of the top 0.1 % differentiated windows out of the total across dogs, wolves and village dogs. Functional elements overlapping the windows are annotated.

Chromosome	Start	End	P-Value	Feature_Chromosome	Feature_Start	Feature_End	Feature_Name	Trait	SV	Protein/Description
Chr2	35943995	35962869	3.6E-07	Chr2	35944004	35946437	ENSCAFT00000009730	Earbend	dups	Uncharacterized, cadherin-like signaling - adhesion. GO:Nervous system development?
Chr2	83935212	84134066	3.6E-07	Chr2	83978926	83979094	ENSCAFT00000026009	Earbend	dups	VPS13D, traficking of membrane proteins, expressed in blood cells - prefrontal cortex
Chr9	17407104	17408535	1.8E-06	Chr9	17407623	17407801	OREG1337672	Earbend	dups	Uncharacterized, homology with ZC3HC1.
ChrUn-AAEX03022357	1000	9900	2.4E-07	ChrUn-AAEX03022357	934	1175	CpG:23	Earbend	dels	-
ChrUn-JH373900	11450	16885	4.8E-07	ChrUn-JH373900	9675	11876	ENSCAFT00000056496	Earbend	dups	lincRNA, blasts well with SRRM3-like protein (splicing machinery)
Chr2	83938683	84134066	1.5E-07	Chr2	83978926	83979094	ENSCAFT00000026009	Boldness	dups	VPS13D, traficking of membrane proteins, expressed in blood cells - prefrontal cortex
Chr5	47659245	47660245	6.2E-07	Chr5	47659167	47659338	ENSCAFT00000046445	Boldness	dups	USP1-201, DNA damage repair
Chr5	47659245	47660245	6.2E-07	Chr5	47659167	47659338	ENSCAFT00000029847	Boldness	dups	USP1-202, DNA damage repair
ChrUn-JH373566	10791	12680	8.3E-07	ChrUn-JH373566	10129	11009	ENSCAFT00000024222	Boldness	dups	Uncharacterized, contains LRRC37AB-C domain. In humans deletion causes neurofibromatosis
Chr16	5790679	5791961	2.6E-10	Chr16	5790120	5791053	ENSCAFT00000024254	Herding	dups	LOC491442, Olfactory receptor
Chr16	5789309	5790679	6.7E-09	Chr16	5790120	5791053	ENSCAFT00000024254	Herding	dups	LOC491442, Olfactory receptor
Chr38	23901851	23903033	2.5E-08	Chr38	23902148	23902214	ENSCAFT00000037740	Herding	dups	Uncharacterized, Rab-GTPase domain
Chr38	23901851	23903033	2.5E-08	Chr38	23902246	23902515	CpG:19	Herding	dups	Uncharacterized, Rab-GTPase domain
Chr21	50754581	50755581	1.3E-07	Chr21	50754899	50754965	ENSCAFT00000047019	Herding	dups	Uncharacterized, Rab-GTPase domain
Chr21	50754581	50755581	1.3E-07	Chr21	50754508	50758008	CpG:260	Herding	dups	Uncharacterized, Rab-GTPase domain
Chr37	148472	149663	5.6E-07	Chr37	148788	148883	ENSCAFT00000031228	Herding	dups	Uncharacterized, Rab-GTPase domain
Chr1	101853329	101855682	1.9E-06	Chr1	101855439	101855614	ENSCAFT00000003948	Herding	dups	Could be NLRP8, inflammosome seems to happen in german shepherds - rottweilers
Chr1	101907810	101909663	1.9E-06	Chr1	101908173	101908204	ENSCAFT00000003974	Herding	dups	NLRP13, inflammosome, seems to happen in german shepherds - rottweilers
Chr1	101898795	101900966	2.0E-06	Chr1	101899834	101899903	ENSCAFT00000003975	Herding	dups	NLRP13, inflammosome, seems to happen in german shepherds - rottweilers
Chr1	101898795	101900966	2.0E-06	Chr1	101899834	101899903	ENSCAFT00000003974	Herding	dups	NLRP13, inflammosome, seems to happen in german shepherds - rottweilers
Chr1	101918381	101921437	2.0E-06	Chr1	101920479	101920650	ENSCAFT00000003975	Herding	dups	NLRP13, inflammosome, seems to happen in german shepherds - rottweilers
Chr1	101918381	101921437	2.0E-06	Chr1	101918221	101918392	ENSCAFT00000003974	Herding	dups	NLRP13, inflammosome, seems to happen in german shepherds - rottweilers
Chr26	12739546	12745570	1.8E-07	Chr26	12744174	12744728	CpG:52	HT	dups	MED13L, really high with our traits
Chr18	48415710	48416710	1.1E-06	Chr18	48415029	48417495	ENSCAFT00000045803	HT	dups	FGF4, associated with height
Chr17	21489286	21490286	1.1E-06	Chr17	21487087	21492444	ENSCAFT00000035869	Legratio	dups	Uncharacterized, homology with GCKR. Diabetes??
Chr19	29820710	29821889	3.1E-07	Chr19	29819829	29821576	ENSCAFT00000007808	Neckratio	dups	RALB, Ras-GTP signaling. Protoncogene, also golgi secretion, in human expressed in blood
Chr16	54943514	54946748	6.0E-07	Chr16	54946699	54947119	CpG:30	Neckratio	dups	No gene, but falls inside a CpG island rich region
Chr9	47308748	47310538	1.1E-06	Chr9	47308011	47308959	ENSCAFT00000030697	Neckratio	dups	Uncharacterized, Olfactory receptor
Chr2	84022658	84023658	8.0E-07	Chr2	84022948	84023079	ENSCAFT00000025995	Shortcoat	dups	VPS13D, traficking of membrane proteins, expressed in blood cells - prefrontal cortex
Chr2	84022658	84023658	8.0E-07	Chr2	84022535	84022711	ENSCAFT00000026009	Shortcoat	dups	VPS13D, traficking of membrane proteins, expressed in blood cells - prefrontal cortex
Chr21	28396851	28398183	1.5E-08	Chr21	28395986	28396937	ENSCAFT00000037933	Tailcurve	dups	LOC485871, Olfactory receptor
Chr16	58993178	58994439	8.8E-07	Chr16	58993962	58994159	ENSCAFT00000038485	Tailcurve	dups	SPAG11, really high in some of our traits, west highland terriers seem to have more CN here
Chr16	59005390	59006712	1.2E-06	Chr16	59006364	59006507	ENSCAFT00000013580	Tailcurve	dups	SPAG11, really high in some of our traits, west highland terriers seem to have more CN here
Chr16	58994439	58996049	2.1E-06	Chr16	58995676	58995731	ENSCAFT00000038485	Tailcurve	dups	SPAG11, really high in some of our traits, west highland terriers seem to have more CN here
Chr35	24544447	24545447	1.2E-07	Chr35	24544082	24544475	ENSCAFT00000043752	Tailratio	dups	Histon related, poorly annotated?
Chr35	24544447	24545447	1.2E-07	Chr35	24543334	24545085	CpG:149	Tailratio	dups	Histon related, poorly annotated?
Chr37	148472	149663	2.0E-07	Chr37	148788	148883	ENSCAFT00000031228	Tailratio	dups	Uncharacterized, Rab-GTPase domain
Chr37	148472	149663	2.0E-07	Chr37	149087	149154	ENSCAFT00000031228	Tailratio	dups	Uncharacterized, Rab-GTPase domain
Chr21	50754581	50755581	1.9E-06	Chr21	50754899	50754965	ENSCAFT00000047019	Tailratio	dups	Uncharacterized, Rab-GTPase domain
Chr21	50754581	50755581	1.9E-06	Chr21	50754508	50758008	CpG:260	Tailratio	dups	Uncharacterized, Rab-GTPase domain
Chr26	12739546	12745570	1.7E-06	Chr26	12744174	12744728	CpG:52	WT	dups	MED13L, really high with our traits
Chr7	43798883	43799883	2.7E-08	Chr7	43798935	43799238	CpG:26	AOD	dels	SMAD2, associated with height, shows linear trend
Chr7	43798883	43799883	2.4E-11	Chr7	43798935	43799238	CpG:26	HT	dels	SMAD2, associated with height, shows linear trend
Chr10	64103282	64105395	6.4E-08	Chr10	64104447	64104746	ENSCAFT00000005113	Tailratio	dels	AFTPH, clathrin mediated traficking of golgi proteins
Chr10	64103282	64105395	6.4E-08	Chr10	64104140	64105096	CpG:90	Tailratio	dels	AFTPH, clathrin mediated traficking of golgi proteins
Chr1	121145033	121146400	1.3E-07	Chr1	121146012	121147002	CpG:70	Tailratio	dels	ZNF536, the region is crowded with CpG islands. In humans represses neuron differentiation
Chr6	15658438	15659828	2.9E-07	Chr6	15652930	15659944	CpG:462	Tailratio	dels	UNCX, homeobox involved in posterior skeleton development, doesn't show linear trend
Chr20	55523407	55525356	3.5E-07	Chr20	55522772	55523480	CpG:51	Tailratio	dels	ZBTB7A, in human represses glycolysis. Tumor supression
Chr20	55523407	55525356	3.5E-07	Chr20	55524049	55524312	CpG:22	Tailratio	dels	ZBTB7A, in human represses glycolysis. Tumor supression
Chr9	19066854	19069031	4.0E-07	Chr9	19066909	19067847	CpG:109	Tailratio	dels	FAM171A2, expressed mainly in brain in adult humans
Chr34	25987376	25989059	4.2E-07	Chr34	25988934	25989158	ENSCAFT00000055812	Tailratio	dels	lincRNA, inside IQCJ-SCHIP1. Probably plays role in neuron transmission
Chr34	25987376	25989059	4.2E-07	Chr34	25988425	25989188	CpG:73	Tailratio	dels	lincRNA, inside IQCJ-SCHIP1. Probably plays role in neuron transmission
Chr7	43798883	43799883	4.8E-12	Chr7	43798935	43799238	CpG:26	WT	dels	SMAD2, associated with height
Chr4	17957526	17959346	3.4E-06	Chr4	17372257	18160293	ENSCAFG00000013221	Cataract	dels	CTNNA3,catenin alpha 3
Chr4	17972161	17975012	3.2E-06	Chr4	17372257	18160293	ENSCAFG00000013221	Cataract	dels	CTNNA3,catenin alpha 3
Chr16	13597554	13600100	2.6E-06	Chr16	13598122	13603063	ENSCAFG00000028887	ElbowDysplasia	dups	-,protein RoBo-1-like
Chr2	84125089	84127691	2.6E-06	Chr2	83912220	84132529	ENSCAFG00000016397	ElbowDysplasia	dups	VPS13D,vacuolar protein sorting 13 homolog D
ChrUn-JH373607	9823	11517	8.9E-07	ChrUn-JH373607	111	15749	ENSCAFG00000016660	ElbowDysplasia	dups	-
Chr18	18447653	18449673	5.0E-06	Chr18	18014357	18699053	ENSCAFG00000004366	HipDysplasia	dups	MAGI2,membrane associated guanylate kinase, WW and PDZ domain containing 2
Chr1	107626224	107630528	4.4E-06	Chr1	107614263	107627150	ENSCAFG00000032038	PatellarLuxation	dels	FUT2,fucosyltransferase 2
Chr1	107626224	107630528	4.4E-06	Chr1	107538972	107628690	ENSCAFG00000003901	PatellarLuxation	dels	BCAT2,branched chain amino acid transaminase 2
Chr1	107626224	107630528	4.4E-06	Chr1	107587250	107642062	ENSCAFG00000003910	PatellarLuxation	dels	RASIP1,Ras interacting protein 1
Chr10	16813598	16814598	4.4E-06	Chr10	16813174	16818015	ENSCAFG00000000561	PatellarLuxation	dels	MIOX,myo-inositol oxygenase
Chr10	16813598	16814598	4.4E-06	Chr10	16813666	16815884	ENSCAFG00000000561	PatellarLuxation	dels	MIOX,myo-inositol oxygenase
Chr13	4021992	4024138	1.5E-06	Chr13	3887464	4023831	ENSCAFG00000000619	PatellarLuxation	dels	UBR5,ubiquitin protein ligase E3 component n-recognin 5
Chr13	34585894	34586894	4.4E-06	Chr13	34579098	35115818	ENSCAFG00000001181	PatellarLuxation	dels	TRAPPC9,trafficking protein particle complex 9
Chr15	29670796	29672473	3.3E-06	Chr15	29587866	29672329	ENSCAFG00000006091	PatellarLuxation	dels	KITLG,KIT ligand
Chr16	14426368	14428811	1.9E-06	Chr16	14407207	14426998	ENSCAFG00000004444	PatellarLuxation	dels	-,zinc finger protein 746
Chr18	49834311	49835496	2.2E-06	Chr18	49826437	49836401	ENSCAFG00000011210	PatellarLuxation	dels	UNC93B1,unc-93 homolog B1, TLR signaling regulator
Chr18	54730309	54731653	6.6E-07	Chr18	54730056	54752646	ENSCAFG00000016070	PatellarLuxation	dels	DAGLA,diacylglycerol lipase alpha
Chr2	8738777	8741979	5.0E-06	Chr2	8741796	8878578	ENSCAFG00000004249	PatellarLuxation	dels	ARHGAP21,Rho GTPase activating protein 21
Chr20	26589110	26590574	5.8E-07	Chr20	26589562	26750109	ENSCAFG00000006777	PatellarLuxation	dels	ADAMTS9,ADAM metallopeptidase with thrombospondin type 1 motif 9
Chr20	52603279	52604279	3.4E-06	Chr20	52594362	52605033	ENSCAFG00000031522	PatellarLuxation	dels	MAP2K7,mitogen-activated protein kinase kinase 7
Chr20	55399156	55401073	4.0E-06	Chr20	55391651	55404603	ENSCAFG00000019121	PatellarLuxation	dels	ANKRD24,ankyrin repeat domain 24
Chr20	55399156	55401073	4.0E-06	Chr20	55383249	55522451	ENSCAFG00000019114	PatellarLuxation	dels	EBI3,Epstein-Barr virus induced 3
Chr20	57163330	57165636	1.7E-07	Chr20	57164743	57181840	ENSCAFG00000019469	PatellarLuxation	dels	ONECUT3,one cut homeobox 3
Chr22	2698582	2700725	3.0E-06	Chr22	2523992	2700213	ENSCAFG00000004379	PatellarLuxation	dels	FNDC3A,fibronectin type III domain containing 3A
Chr28	33809998	33811556	1.9E-06	Chr28	33806214	33811331	ENSCAFG00000031292	PatellarLuxation	dels	NKX1-2,NK1 homeobox 2
Chr28	36882410	36883410	3.4E-08	Chr28	36746821	36902083	ENSCAFG00000013204	PatellarLuxation	dels	PTPRE,protein tyrosine phosphatase, receptor type E
Chr38	22125713	22126897	4.0E-07	Chr38	22114144	22140086	ENSCAFG00000029844	PatellarLuxation	dels	-
Chr4	51589903	51590903	5.0E-07	Chr4	51379232	51765864	ENSCAFG00000017318	PatellarLuxation	dels	EBF1,EBF transcription factor 1
Chr5	6095603	6096603	2.1E-06	Chr5	6073009	6185948	ENSCAFG00000010304	PatellarLuxation	dels	ETS1,ETS proto-oncogene 1, transcription factor
Chr5	40827582	40828767	3.6E-06	Chr5	40828284	40832707	ENSCAFG00000030269	PatellarLuxation	dels	MFAP4,microfibril associated protein 4
Chr8	47468460	47469460	4.7E-07	Chr8	47455429	47473918	ENSCAFG00000016913	PatellarLuxation	dels	VSX2,visual system homeobox 2
Chr8	72331785	72332785	4.6E-06	Chr8	72315825	72335651	ENSCAFG00000018354	PatellarLuxation	dels	AKT1,AKT serine/threonine kinase 1
Chr9	10685742	10687960	4.6E-06	Chr9	10686538	10687504	ENSCAFG00000030037	PatellarLuxation	dels	-
Chr9	19066854	19069031	3.7E-06	Chr9	19067276	19111341	ENSCAFG00000014156	PatellarLuxation	dels	FAM171A2,family with sequence similarity 171 member A2
Chr9	21066378	21067378	4.6E-06	Chr9	21065845	21076123	ENSCAFG00000015920	PatellarLuxation	dels	HAP1,huntingtin associated protein 1
Chr9	21066378	21067378	4.6E-06	Chr9	21066668	21209343	ENSCAFG00000015952	PatellarLuxation	dels	KRT9,keratin 9
Chr9	24803596	24804822	4.8E-07	Chr9	24803208	24803880	ENSCAFG00000031634	PatellarLuxation	dels	-
Chr9	24803596	24804822	4.8E-07	Chr9	24804425	24806296	ENSCAFG00000016843	PatellarLuxation	dels	HOXB2,homeobox B2
Chr13	34585894	34586894	3.1E-06	Chr13	34579098	35115818	ENSCAFG00000001181	RetinalAtrophy	dels	TRAPPC9,trafficking protein particle complex 9
Chr16	59006712	59009382	1.2E-06	Chr16	59006364	59011878	ENSCAFG00000008558	RetinalAtrophy	dups	SPAG11B,sperm associated antigen 11B
Chr28	32227459	32228459	4.8E-06	Chr28	32197969	32286584	ENSCAFG00000012561	RetinalAtrophy	dups	DMBT1,deleted in malignant brain tumors 1
Chr28	32242199	32243199	3.5E-06	Chr28	32197969	32286584	ENSCAFG00000012561	RetinalAtrophy	dups	DMBT1,deleted in malignant brain tumors 1
Chr7	65816718	65817718	3.4E-06	Chr7	65794554	65962531	ENSCAFG00000018235	Thyroid	dels	-

Supplemental Table S2 – GWAS significant results for the all assayed traits above secondary p-value threshold overlapping annotations in CanFam3.1 and the ORegAnno database for dogs.

Trait code name	Trait full name	Type of variable (range)	Description	Test used	Number of significant windows	Trait Source	Included (reason)
Aggressiveness	Aggressiveness	Continuous score (1.5-2.5)	-	Categorical Stratified CMH (two strata)	0	(Plassais et al. 2019)	NO (excess missing)
AOD	Age of death	Continuous measure	Average breed age of death (in years)	Linear Regression (PCA corrected)	8	(Jones et al. 2008)	YES
AttachmentAttentionSeeking	Attachment attention seeking	Continuous score (0-4)	Maintaining close proximity to the owner or other members of the household, soliciting affection or attention, and displaying agitation when the owner gives attention to third parties	Categorical Stratified CMH (two strata)	0	(McLeant et al. 2019)	YES
BodyheightF	Height at the withers females	Continuous measure	Average breed height at the withers for females (in cm)	Linear Regression (PCA corrected)	115	ours	NO (redundant to Plassais)
BodyheightM	Height at the withers males	Continuous measure	Average breed height at the withers for males (in cm)	Linear Regression (PCA corrected)	89	ours	NO (redundant to Plassais)
BodyweightF	Weight females	Continuous measure	Average breed weight for females (in kg)	Linear Regression (PCA corrected)	52	ours	NO (redundant to Plassais)
BodyweightM	Weight males	Continuous measure	Average breed weight for males (in kg)	Linear Regression (PCA corrected)	33	ours	NO (redundant to Plassais)
Boldness	Boldness	Categorical variable (2 factors)	-	Categorical Stratified CMH (two strata)	0	(Plassais et al. 2019)	NO (excess missing)
Boldness	Boldness	Categorical variable (2 factors)	Dog trainer assessment of breed bolness	Categorical Stratified CMH (two strata)	2	(Jones et al. 2008)	YES
Bulky	Bulky	Categorical variable (2 factors)	Characterized heavy muscling and back fat thickness which is observed in 54% of the large dog breeds	Categorical Stratified CMH (two strata)	34	(Plassais et al. 2019)	YES
Cardiac	Cardiac disease propensity	Rank variable	Ranking of dog breeds based on the normalized number of heart related disease diagnostics	Categorical non-stratified CMH	0	OFA	YES
Cataract	Cataract disease propensity	Rank variable	Ranking of dog breeds based on the normalized number of cataract diagnostics	Categorical Stratified CMH (two strata)	0	OFA	YES
Chasing	Chasing	Continuous score (0-4)	Chasing cats, birds, and/or other small animals, given the opportunity	Categorical non-stratified CMH	0	(McLeant et al. 2019)	YES
chest_rat_mn	Chest to body ratio	Percentage (0-1)	Average breed chest to body ratio (see reference)	Categorical non-stratified CMH	13	(Jones et al. 2008)	YES
DogDirectedAggression	Dog directed aggression	Continuous score (0-4)	Threatening or hostile responses when approached by unfamiliar dogs	Categorical non-stratified CMH	7	(McLeant et al. 2019)	YES
DogDirectedFear	Dog directed fear	Continuous score (0-4)	Fearful or wary responses when approached by unfamiliar dogs	Categorical non-stratified CMH	0	(McLeant et al. 2019)	YES
DogRivalry	Dog rivalry	Continuous score (0-4)	Threatening or hostile responses to other familiar dogs in the same household	Categorical Stratified CMH (two strata)	0	(McLeant et al. 2019)	YES
Dropears	Drop ears	Categorical variable (2 factors)	Does the dog have hanging ears (1:no, 2:yes)?	Categorical non-stratified CMH	22	(Plassais et al. 2019)	YES
Earbend	Ear bend	Categorical variable (4 factors)	Breed ear bend scaled from 1 (hanging low) to 4 (completely erect)	Categorical non-stratified CMH	31	(Jones et al. 2008)	YES
Ectropion	Ectropion disease propensity	Rank variable	Ranking of dog breeds based on the normalized number of ectopion diagnostics	-	0	OFA	NO (excess missing)
ElbowDysplasia	Elbow dysplasia	Rank variable	Ranking of dog breeds based on the normalized number of elbow dysplasia diagnostics	Categorical non-stratified CMH	6	OFA	YES
EnergyLevel	Energy level	Continuous score (0-4)	Energetic, "always on the go", and/or playful	Categorical non-stratified CMH	6	(McLeant et al. 2019)	YES
excitability	Excitability	Discrete (ordinal) variable (10 factors)	Data on how excitable a breed is taken from Hart and Miller (1985)	Categorical Stratified CMH (two strata)	0	(Jones et al. 2008)	YES
Excitability	Excitability	Continuous score (0-4)	Displaying strong reactions to potentially exciting or arousing events, such as going for walks or car trips, doorbells, arrival of visitors, and the owner arriving home; has difficulty settling down after such events	Categorical non-stratified CMH	15	(McLeant et al. 2019)	YES
Fighting	Fighting	Categorical variable (2 factors)	Is the dog breed commonly used in dog fights?	Categorical non-stratified CMH	0	ours	YES
Furnish	Furnish	Categorical variable (2 factors)	Do the breeds have furnishings (mustache, beard, or eyebrows) (1:no, 2:yes)?	Categorical Stratified CMH (two strata)	22	(Plassais et al. 2019)	YES
Hairlength	Hairlength	Discrete (ordinal) variable (4 factors)	Breed standard report of hair length categories are Long, Medium, Short and Hairless	Categorical non-stratified CMH	72	ours	YES
Hairless	Hairless	Categorical variable (2 factors)	Is the dog breed hairless?	Categorical Stratified CMH (two strata)	0	(Plassais et al. 2019)	YES
Headratio	Head ratio	Percentage (0-1)	Average breed head to body ratio (see reference)	Categorical Stratified CMH (two strata)	0	(Jones et al. 2008)	YES
Height	Height	Continuous measure	Average breed height at the withers (in in)	Linear Regression (PCA corrected)	10	(Plassais et al. 2019)	YES
Herding	Herding	Categorical variable (2 factors)	Is the dog breed commonly used for herding animals?	Categorical non-stratified CMH	23	(Jones et al. 2008)	YES
HipDysplasia	Hip dysplasia propensity	Rank variable	Ranking of dog breeds based on the normalized number of elbow dysplasia diagnostics	Categorical non-stratified CMH	0	OFA	YES
HT	Height at the withers	Continuous measure	Average breed height at the withers (in in)	Categorical non-stratified CMH	61	(Jones et al. 2008)	NO (redundant to Plassais)
IgA	Immunoglobulin A production	Continuous score (0.08-0.75)	Breed average ELISA IgA titration	-	0	(Olsson et al. 2014)	NO (excess missing)

(continuation)

Trait code name	Trait full name	Type of variable (range)	Description	Test used	Number of significant windows	Trait Source	Included (reason)
Intelligence	Intelligence	Discrete (ordinal) variable (6 factors)	Breed score on memory and trainability (see reference)	Categorical non-stratified CMH	0	ours	YES
Largeears	Large ears	Categorical variable (2 factors)	Does the breed have characteristically large ears (1:no, 2:yes)?	Categorical non-stratified CMH	25	(Plassais et al. 2019)	YES
Legratio	Leg ratio	Percentage (0-1)	Average breed leg to body ratio (see reference)	Categorical Stratified CMH (two strata)	1	(Jones et al. 2008)	YES
Lenghoffur	Lengh of fur	Categorical variable (2 factors)	Report of fur length (1:short, 2:long)	Categorical non-stratified CMH	5	(Plassais et al. 2019)	YES
Lifespan	Lifespan	Continuous measure	Average breed age of death (in years)	Linear Regression (PCA corrected)	5	(Plassais et al. 2019)	YES
Lifespan	Lifespan	Continuous measure	Average breed age of death (in years)	Linear Regression (PCA corrected)	7	ours	YES
Littersize	Litter size	Continuous measure	Average breed litter count	Linear Regression (PCA corrected)	74	(Borge et al. 2011)	YES
Longlegs	Long legs	Categorical variable (2 factors)	Is the breed considered a sighthound (1:no, 2:yes)?	-	2	(Plassais et al. 2019)	NO (excess missing)
Muscled	Muscled	Categorical variable (2 factors)	Similar to the bulky phenotype but considering small breeds as well	Categorical non-stratified CMH	4	(Plassais et al. 2019)	YES
Neckratio	Neck to body ratio	Percentage (0-1)	Average breed neck to body ratio (see reference)	Categorical non-stratified CMH	6	(Jones et al. 2008)	YES
NonsocialFear	Nonsocial fear	Continuous score (0-4)	Fearful or wary responses to sudden or loud noises, traffic, and unfamiliar objects and situations	Categorical Stratified CMH (two strata)	0	(McLeant et al. 2019)	YES
OwnerDirectedAggression	Owner directed aggression	Continuous score (0-4)	Threatening or hostile responses to the owner or other members of the household when challenged, manhandled, stared at, stepped over, or when approached while in possession of food or objects	Categorical non-stratified CMH	0	(McLeant et al. 2019)	YES
PatellarLuxation	Patellar luxation propensity	Rank variable	Ranking of dog breeds based on the normalized number of patellar luxation diagnostics	Categorical non-stratified CMH	4	OFA	YES (close to excess missing)
RetinalAtrophy	Retinal atrophy (generalized) propensity	Rank variable	Ranking of dog breeds based on the normalized number of generalized retinal atrophy diagnostics	Categorical non-stratified CMH	1	OFA	YES
SeparationRelatedBehavior	Separation related behavior	Continuous score (0-4)	Vocalizing and/or destructiveness when separated from the owner, often accompanied or preceded by behavioral and autonomic signs of anxiety including restlessness, loss of appetite, trembling, and excessive salivation	Categorical Stratified CMH (two strata)	3	(McLeant et al. 2019)	YES
Shortcoat	Short coat	Categorical variable (2 factors)	Coded with 1 for breeds with a very short coat and 0 for the rest	Categorical non-stratified CMH	1	(Jones et al. 2008)	YES
SighthoundRacing	Sighthound racing	Categorical variable (2 factors)	Is the dog breed used in dog races/has a sighthound phenotype?	Categorical Stratified CMH (two strata)	1	ours	YES
Smell	Smell	Categorical variable (2 factors)	Is the dog breed commonly used for smell detection (explosives, drugs ...)	Categorical Stratified CMH (two strata)	0	ours	YES
Snoutangle	Snout angle	Continuous score (75-209)	Average breed angle from muzzle to stop	Categorical non-stratified CMH	0	(Jones et al. 2008)	YES
Snoutratio	Snout to body ratio	Percentage (0-1)	Average breed snout to body ratio (see reference)	Categorical non-stratified CMH	0	(Jones et al. 2008)	YES
StrangerDirectedAggression	Stranger directed aggression	Continuous score (0-4)	Threatening or hostile responses to strangers approaching or invading the dog's or owner's personal space, territory, or home range	Categorical non-stratified CMH	0	(McLeant et al. 2019)	YES
StrangerDirectedFear	Stranger directed fear	Continuous score (0-4)	Fearful or wary responses when approached by strangers	Categorical Stratified CMH (two strata)	0	(McLeant et al. 2019)	YES
Tailcurl	Tail curl	Discrete (ordinal) variable (4 factors)	Breed degree of curve in the tail on a scale of 1 (straight) to 4 (tightly curled)	Categorical Stratified CMH (two strata)	0	(Plassais et al. 2019)	YES (close to excess missing)
Tailcurve	Tail curve	Discrete (ordinal) variable (5 factors)	Breed degree of curve in the tail on a scale of 1 (straight) to 5 (tightly curled)	Categorical Stratified CMH (two strata)	17	(Jones et al. 2008)	YES
Tailratio	Tail to body ratio	Percentage (0-1)	Average breed tail to body ratio (see reference)	Categorical non-stratified CMH	9	(Jones et al. 2008)	YES
Temperament	Temperament	Continuous score (0-100)	Average breed score on obedience and command memmorization (see reference)	Categorical Stratified CMH (two strata)	0	ATTS	YES
Thyroid	Thyroid	Rank variable	Ranking of dog breeds based on the normalized number of thyroid related disease diagnostics	Categorical non-stratified CMH	0	OFA	YES (close to excess missing)
TouchSensitivity	Touch sensitivity	Continuous score (0-4)	Fearful or wary responses to potentially painful procedures, including bathing, grooming, nail-clipping, and veterinary examinations	Categorical Stratified CMH (two strata)	5	(McLeant et al. 2019)	YES
Trainability	Trainability	Categorical variable (2 factors)	Dog trainer assessment of how easily a breed can be trained	Categorical non-stratified CMH	1	(Jones et al. 2008)	YES
Trainability	Trainability	Continuous score (0-4)	Willingness to attend to the owner, obey simple commands, learn quickly, fetch objects, respond positively to correction, and ignore distracting stimuli	Categorical non-stratified CMH	2	(McLeant et al. 2019)	YES
Weight	Weight	Continuous measure	Average breed weight (in lb)	Linear Regression (PCA corrected)	15	(Plassais et al. 2019)	YES
Whitechest	White chest	Categorical variable (2 factors)	Is the breed's chest fur white?	Categorical non-stratified CMH	0	(Plassais et al. 2019)	YES
Whitehead	White head	Categorical variable (2 factors)	Is the breed's head fur white?	Categorical Stratified CMH (two strata)	0	(Plassais et al. 2019)	YES (close to excess missing)
WT0.33resid	Residual of weight to lifespan regression	Continuous score (-0.67-0.79)	Regression residual of breed average lifespan vs weight	Linear Regression (PCA corrected)	45	(Jones et al. 2008)	YES
WT	Weight	Continuous measure	Average breed weight (in lb)	Linear Regression (PCA corrected)	14	(Jones et al. 2008)	NO (redundant to Plassais)

Supplemental Table S3 – Description of GWAS phenotypes, number of association signals and test performed.

Non-Exonic CNV duplications	Conserved	Non-Conserved	
Associated	8	53	P-val=0.38
Non-associated	394	3680	

Non-Exonic CNV Deletions	Conserved	Non-Conserved	
Associated	51	238	P-val=0.15
Non-associated	10630	39278	

Non-Exonic CNV all	Conserved	Non-Conserved	
Associated	59	281	P-val=0.15
Non-associated	10931	42214	

Supplemental Table S4 – Contingency table of conserved elements inside and outside associated windows.

Chromosome	Start	End	Ensembl_transcript	Feature_name	Trait
Chr26	11696564	11708211	ENSCAFT00000014674	TBX3	BodyheightF
Chr7	44336287	44363163	ENSCAFT00000027863	HDHD2	BodyheightF
Chr7	44370296	44413497	ENSCAFT00000027875	KATNAL2	BodyheightF
Chr7	44309588	44363129	ENSCAFT00000035344	HDHD2	BodyheightF
Chr7	44338091	44437346	ENSCAFT00000042868	KATNAL2	BodyheightF
Chr7	44372262	44412793	ENSCAFT00000044953	KATNAL2	BodyheightF
Chr7	44370299	44587494	ENSCAFT00000045108	KATNAL2	BodyheightF
Chr26	11697220	11708211	ENSCAFT00000046183	TBX3	BodyheightF
Chr26	11666482	11667175	ENSCAFT00000053431	lincRNA	BodyheightF
Chr26	11696564	11708211	ENSCAFT00000014674	TBX3	BodyheightM
Chr7	44336287	44363163	ENSCAFT00000027863	HDHD2	BodyheightM
Chr7	44370296	44413497	ENSCAFT00000027875	KATNAL2	BodyheightM
Chr7	44309588	44363129	ENSCAFT00000035344	HDHD2	BodyheightM
Chr17	36199018	36202608	ENSCAFT00000035694	protein_coding	BodyheightM
Chr7	44338091	44437346	ENSCAFT00000042868	KATNAL2	BodyheightM
Chr7	44372262	44412793	ENSCAFT00000044953	KATNAL2	BodyheightM
Chr7	44370299	44587494	ENSCAFT00000045108	KATNAL2	BodyheightM
Chr26	11697220	11708211	ENSCAFT00000046183	TBX3	BodyheightM
Chr17	36165207	36167595	ENSCAFT00000051403	lincRNA	BodyheightM
Chr17	36178287	36180035	ENSCAFT00000052000	lincRNA	BodyheightM
Chr17	35841306	36076836	ENSCAFT00000052207	lincRNA	BodyheightM
Chr26	11666482	11667175	ENSCAFT00000053431	lincRNA	BodyheightM
Chr17	36009692	36127383	ENSCAFT00000053686	lincRNA	BodyheightM
Chr17	35943579	35945825	ENSCAFT00000056000	lincRNA	BodyheightM
Chr26	11696564	11708211	ENSCAFT00000014674	TBX3	BodyweightF
Chr7	44336287	44363163	ENSCAFT00000027863	HDHD2	BodyweightF
Chr7	44370296	44413497	ENSCAFT00000027875	KATNAL2	BodyweightF
Chr7	44309588	44363129	ENSCAFT00000035344	HDHD2	BodyweightF
Chr7	44338091	44437346	ENSCAFT00000042868	KATNAL2	BodyweightF
Chr7	44372262	44412793	ENSCAFT00000044953	KATNAL2	BodyweightF
Chr7	44370299	44587494	ENSCAFT00000045108	KATNAL2	BodyweightF
Chr26	11697220	11708211	ENSCAFT00000046183	TBX3	BodyweightF
Chr26	11666482	11667175	ENSCAFT00000053431	lincRNA	BodyweightF
Chr26	11696564	11708211	ENSCAFT00000014674	TBX3	BodyweightM
Chr7	44336287	44363163	ENSCAFT00000027863	HDHD2	BodyweightM
Chr7	44370296	44413497	ENSCAFT00000027875	KATNAL2	BodyweightM
Chr7	44309588	44363129	ENSCAFT00000035344	HDHD2	BodyweightM
Chr17	36199018	36202608	ENSCAFT00000035694	protein_coding	BodyweightM
Chr7	44338091	44437346	ENSCAFT00000042868	KATNAL2	BodyweightM
Chr7	44372262	44412793	ENSCAFT00000044953	KATNAL2	BodyweightM
Chr7	44370299	44587494	ENSCAFT00000045108	KATNAL2	BodyweightM
Chr26	11697220	11708211	ENSCAFT00000046183	TBX3	BodyweightM
Chr17	36165207	36167595	ENSCAFT00000051403	lincRNA	BodyweightM
Chr17	36178287	36180035	ENSCAFT00000052000	lincRNA	BodyweightM
Chr17	35841306	36076836	ENSCAFT00000052207	lincRNA	BodyweightM
Chr26	11666482	11667175	ENSCAFT00000053431	lincRNA	BodyweightM
Chr17	36009692	36127383	ENSCAFT00000053686	lincRNA	BodyweightM
Chr17	35943579	35945825	ENSCAFT00000056000	lincRNA	BodyweightM
Chr33	785672	1092293	ENSCAFT00000014024	EPHA3	Hairlength
Chr37	6432400	6499256	ENSCAFT00000017095	PGAP1	Hairlength
Chr9	15840678	15962428	ENSCAFT00000017124	MAP2K6	Hairlength
Chr9	17334729	17340976	ENSCAFT00000021268	protein_coding	Hairlength
Chr33	1129049	1129156	ENSCAFT00000040553	RF00019	Hairlength
Chr9	17335270	17341774	ENSCAFT00000043194	protein_coding	Hairlength
Chr37	6423290	6499361	ENSCAFT00000043813	PGAP1	Hairlength
Chr9	17335270	17340910	ENSCAFT00000050102	protein_coding	Hairlength
Chr33	1143849	1149164	ENSCAFT00000052066	lincRNA	Hairlength
Chr33	1126850	1128113	ENSCAFT00000053824	lincRNA	Hairlength
Chr23	18984614	19058869	ENSCAFT00000057582	lincRNA	Hairlength
Chr23	19032257	19058869	ENSCAFT00000057649	lincRNA	Hairlength
Chr23	19032506	19103277	ENSCAFT00000059093	lincRNA	Hairlength
Chr33	1147978	1150689	ENSCAFT00000059143	lincRNA	Hairlength
Chr12	65087461	65120744	ENSCAFT00000006043	OSTM1	Intelligence
Chr4	64777068	65160441	ENSCAFT00000029386	HCN1	Intelligence
Chr1	7926235	7931932	ENSCAFT00000044917	protein_coding	Intelligence
Chr4	64776432	65163127	ENSCAFT00000049582	HCN1	Intelligence
Chr7	44336287	44363163	ENSCAFT00000027863	HDHD2	Lifespan
Chr7	44370296	44413497	ENSCAFT00000027875	KATNAL2	Lifespan
Chr7	44309588	44363129	ENSCAFT00000035344	HDHD2	Lifespan
Chr7	44338091	44437346	ENSCAFT00000042868	KATNAL2	Lifespan
Chr7	44372262	44412793	ENSCAFT00000044953	KATNAL2	Lifespan
Chr7	44370299	44587494	ENSCAFT00000045108	KATNAL2	Lifespan
Chr7	44336287	44363163	ENSCAFT00000027863	HDHD2	Littersize
Chr7	44370296	44413497	ENSCAFT00000027875	KATNAL2	Littersize
Chr7	44309588	44363129	ENSCAFT00000035344	HDHD2	Littersize
Chr7	44338091	44437346	ENSCAFT00000042868	KATNAL2	Littersize
Chr7	44372262	44412793	ENSCAFT00000044953	KATNAL2	Littersize
Chr7	44370299	44587494	ENSCAFT00000045108	KATNAL2	Littersize

Supplemental Table S5 – Hi-C contacts containing associated genes.

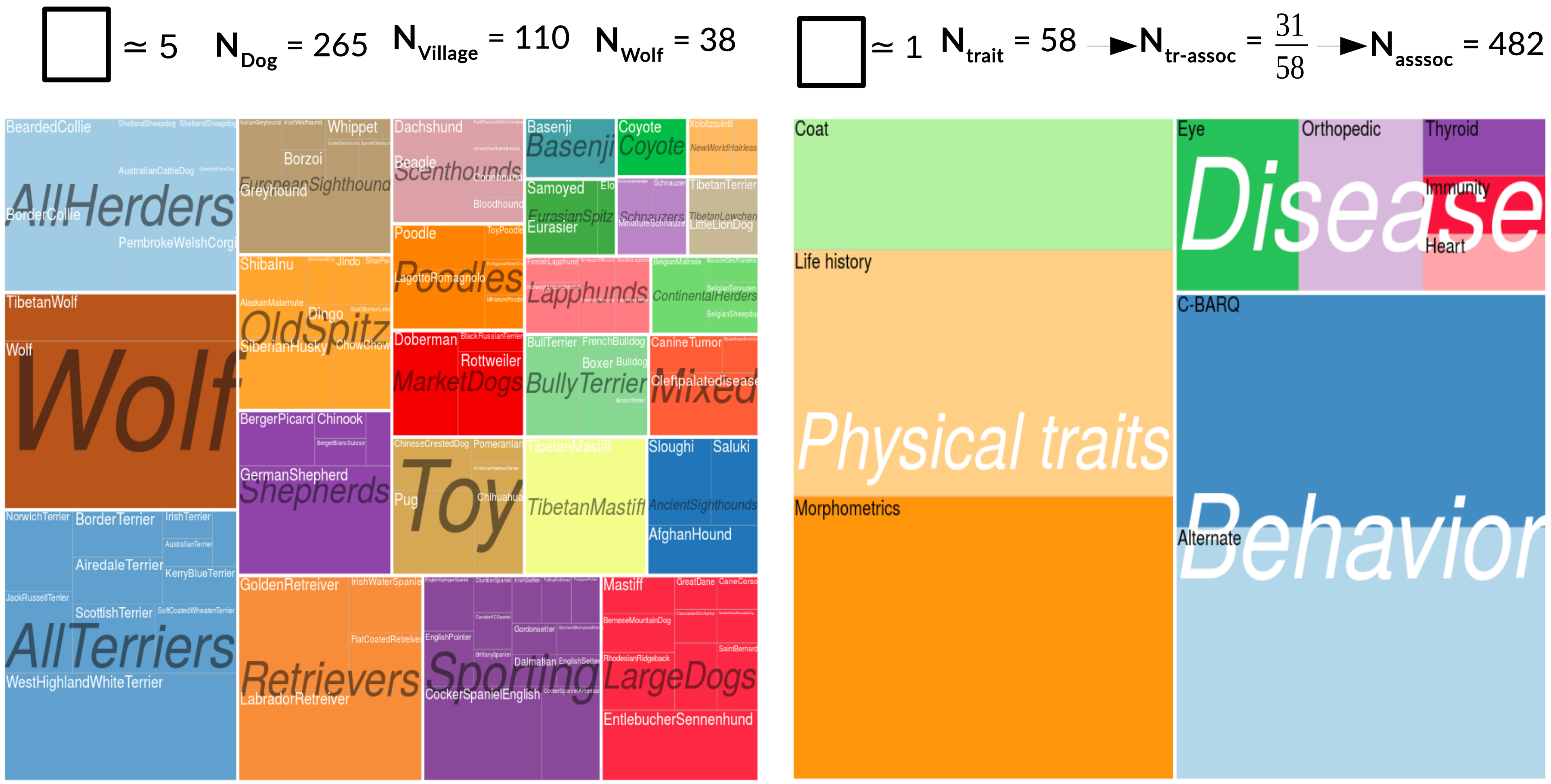

Supplemental Figure S1 – Visual representation of sample and phenotype numbers. Left: samples partitioned by breed and clade; on the top is a square representing the number of samples per area unit and the absolute number of dogs, village dogs and wolves. Right: phenotypes partitioned by type; on the top is a square representing the number of traits per area unit and the absolute number of traits, the fraction of traits with at least one CNV association and the total number of CNV asociations across all traits.

	Original panel		aCGH panel	
**Data**	WGS	301	Nimblegen2M	64
			Nimblegen100K	64
			NimblegenCustom	14
**Species**	Breed(#)	263 (~100)	Breed(#)	128 (32)
	Wolves	38	Wolves	14

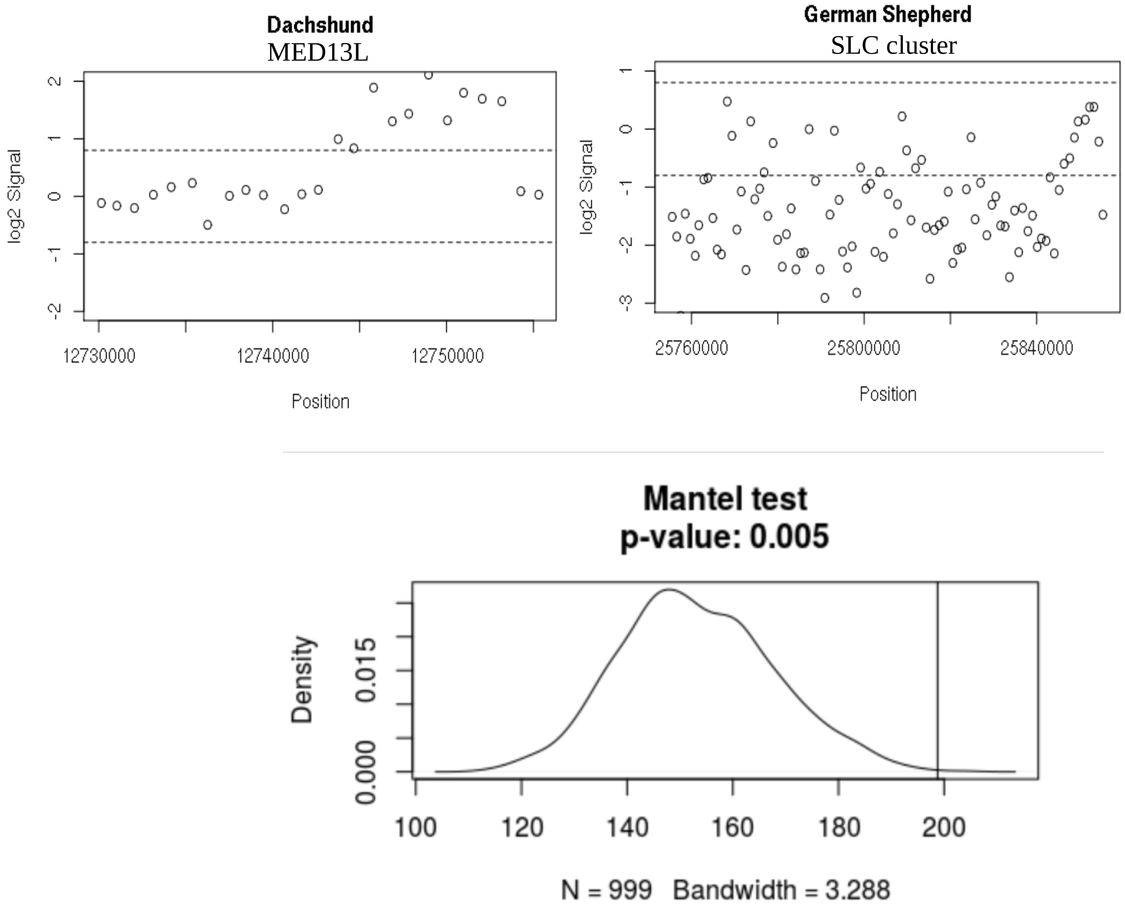

Supplemental Figure S2 – The frequencies of 12 CNV loci were assessed in 32 breeds common between our WGS panel and an array comparative genomics (aCGH) cohort (Ramirez et al. 2014; Nicholas et al. 2011; Berglund et al. 2012).
Top: table of the samples used for validation. The total number of samples and breeds in the WGS and the aCGH panels is represented.
Middle: two examples of validated CNVs with concordant gain-loss status in WGS and aCGH. aCGH probe signal intensity is plotted in the y axis against probe coordinates. Per-breed CNV frequencies were determined based on gain-loss concordance as depicted in these examples.
Bottom: global validation of CNV locus frequencies per breed. The similarity between frequency matrices resulting from both platforms was assessed using a mantel test. Per locus concordance was also assessed via correlation (data not shown).

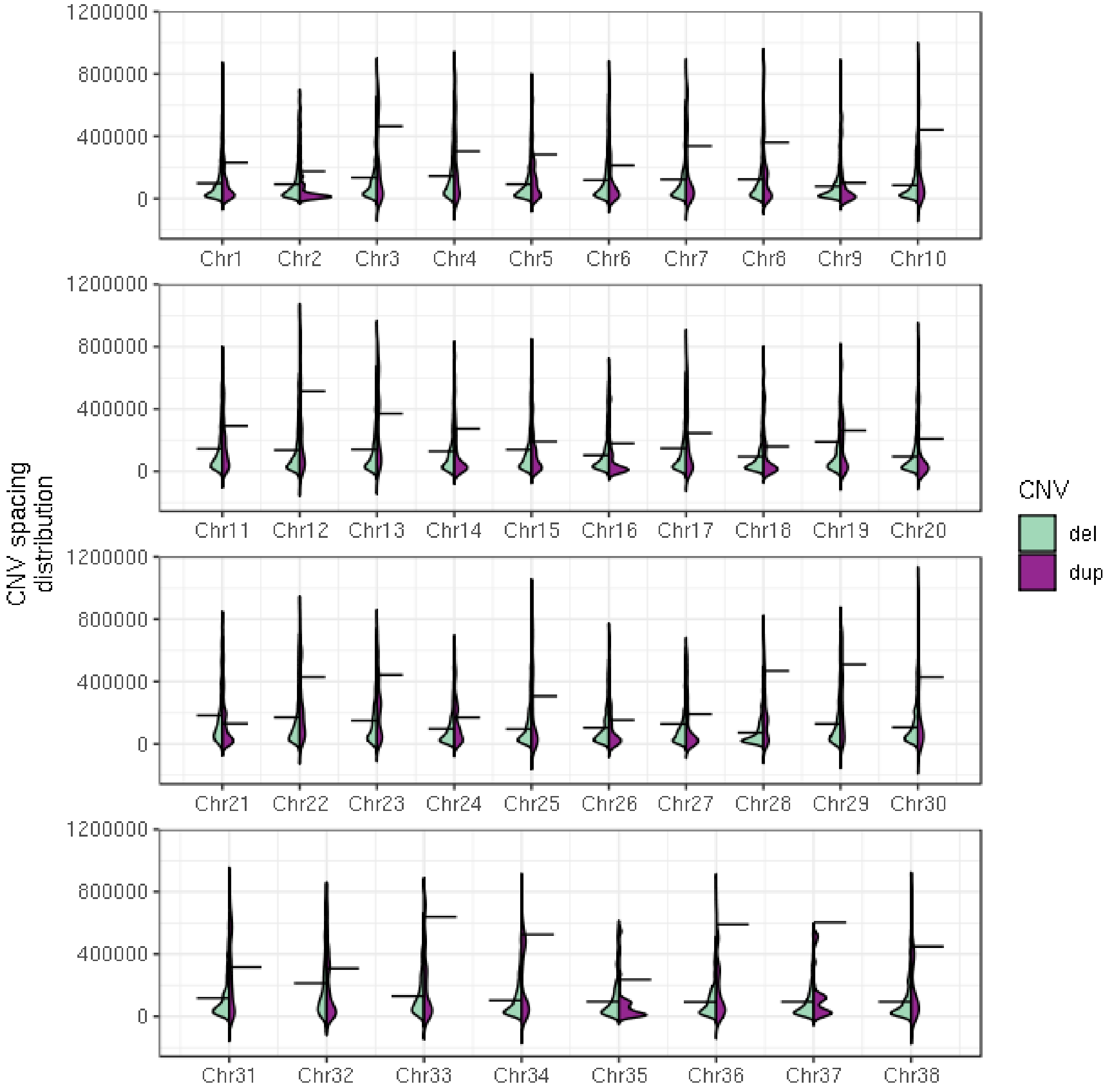

Supplemental Figure S3 – Violin plots of CNV distances per dog chromosome. The distributions of duplications and deletions are colored purple and turquoise respectively. The protruding lines on each violin plot correspond to the expected distance between events if they were evenly distributed along a chromosome. They were calculated by dividing the available chromosome space (length of the chromosome minus length of repeats and CNVs within that chromosome) by the number of CNV events in each respective chrmosome. The expected distance between duplications is significantly larger than expected under an exponential distribution of our data points, meaning that duplications tend to cluster. The expected distance between deletions is not significant. (Bonferroni-corrected, Fisher-aggregated exponential distribution test for expected distance $\text{p-value}_{duplications}$=0.006 and $\text{p-value}_{deletions}$=0.9).

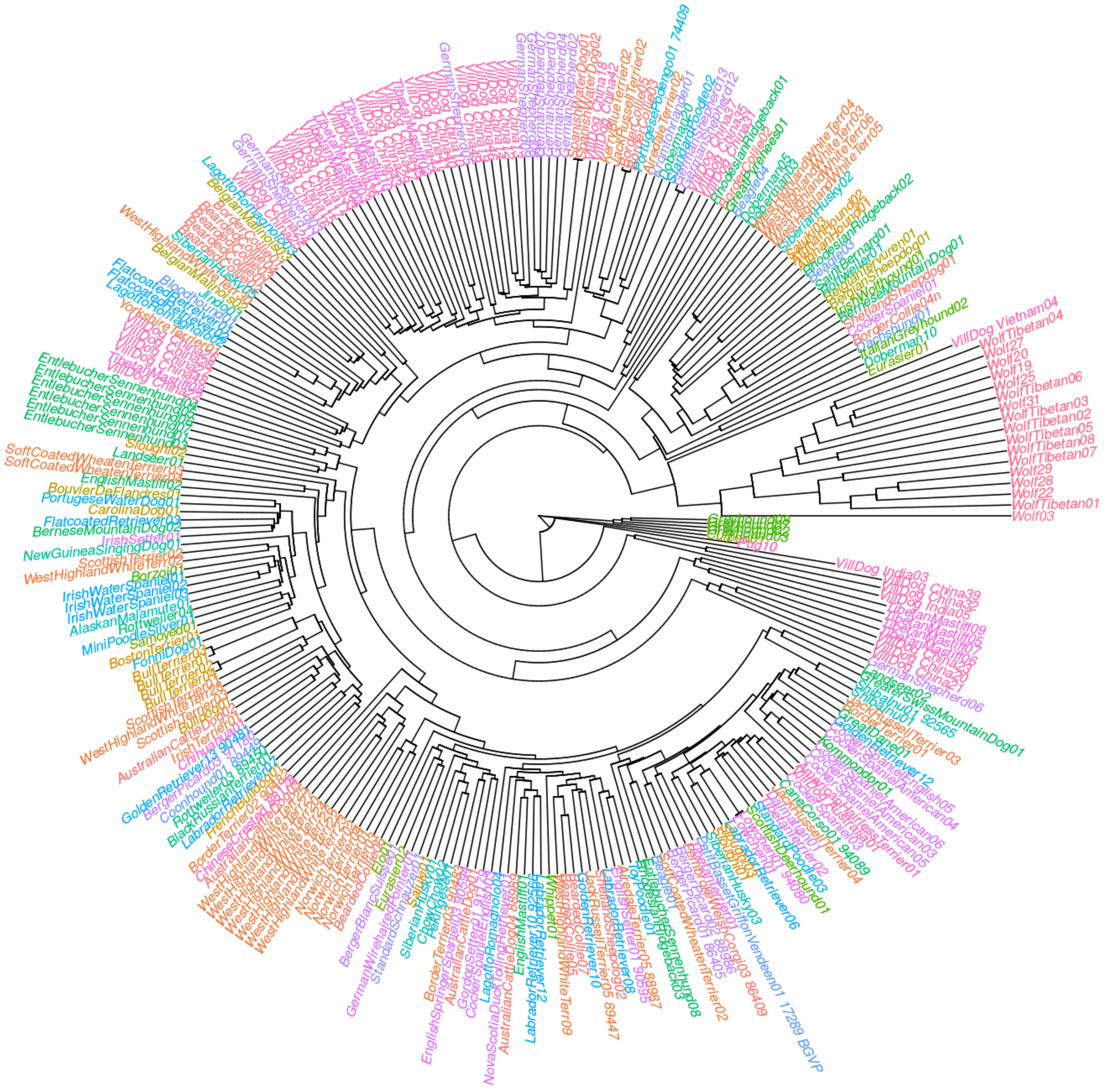

Supplemental Figure S4 – Breed phylogeny using CNVs. Breed macrogroups are colored accordingly. Contrary to haplogroup and IBD trees, we observe sparse clustering by macrogroup aside from individuals belonging to the same breed, pointing to an unstratified nature of the data.

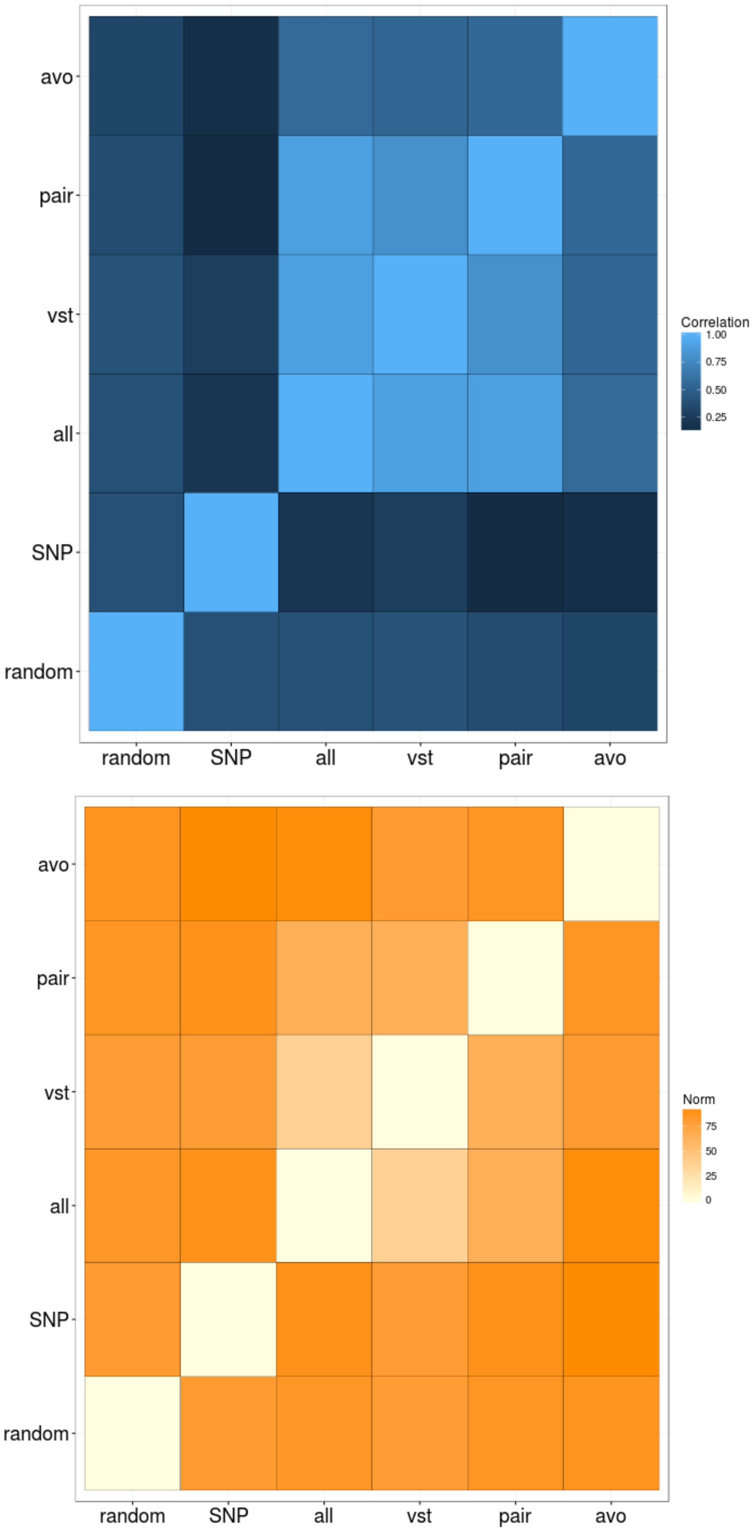

Supplemental Figure S5 – Breed phylogeny CNV comparison to Haplotype based trees. Random: randomly generated distance matrix (zero similarity); SNP: Haplotype based distance matrix; all: distance matrix using all structural variation; vst: distance matrix using only breed-differentiated structural variants; pair: distance matrix using all structural variants but excluding breed-admixed haplotypes for each pair of breeds. avo: distance matrix using all structural variants but excluding any breed-admixed haplotypes across all breeds. Top/blue: distance matrix correlation; Bottom/orange: 2-norm scaled matrix distance. On a global scale, we see little similarity to the basic SNP tree topology.

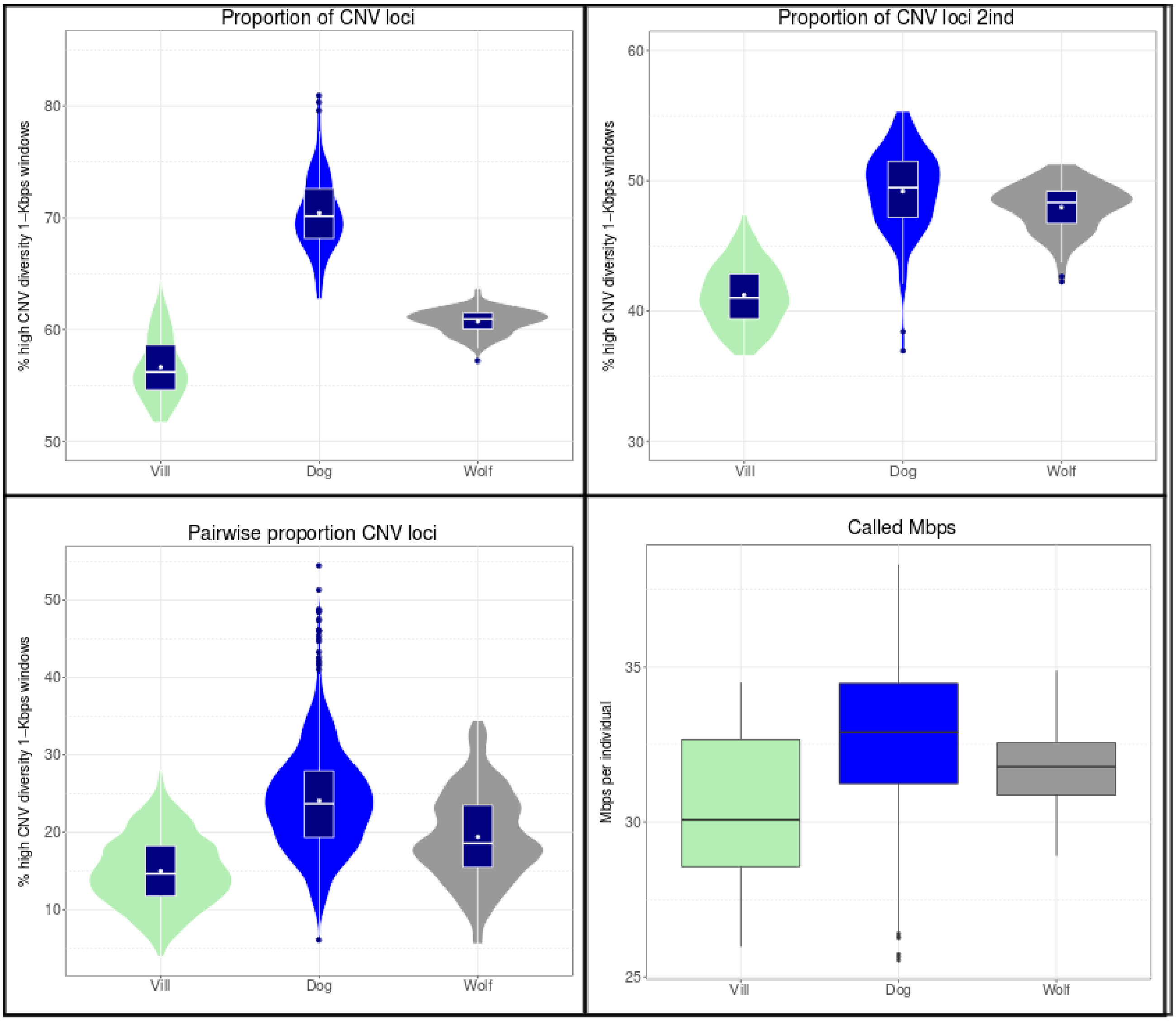

Supplemental Figure S6 – Proportion of segregating copy number variants in village dogs (green), breed dogs (blue) and wolves (gray). Top left: raw proportions; Top right: proportions using CNV present in three or more individuals ; Bottom left: pairwise proportions of segregating CNVs; bottom right raw amount of copy number calls per subspecies.

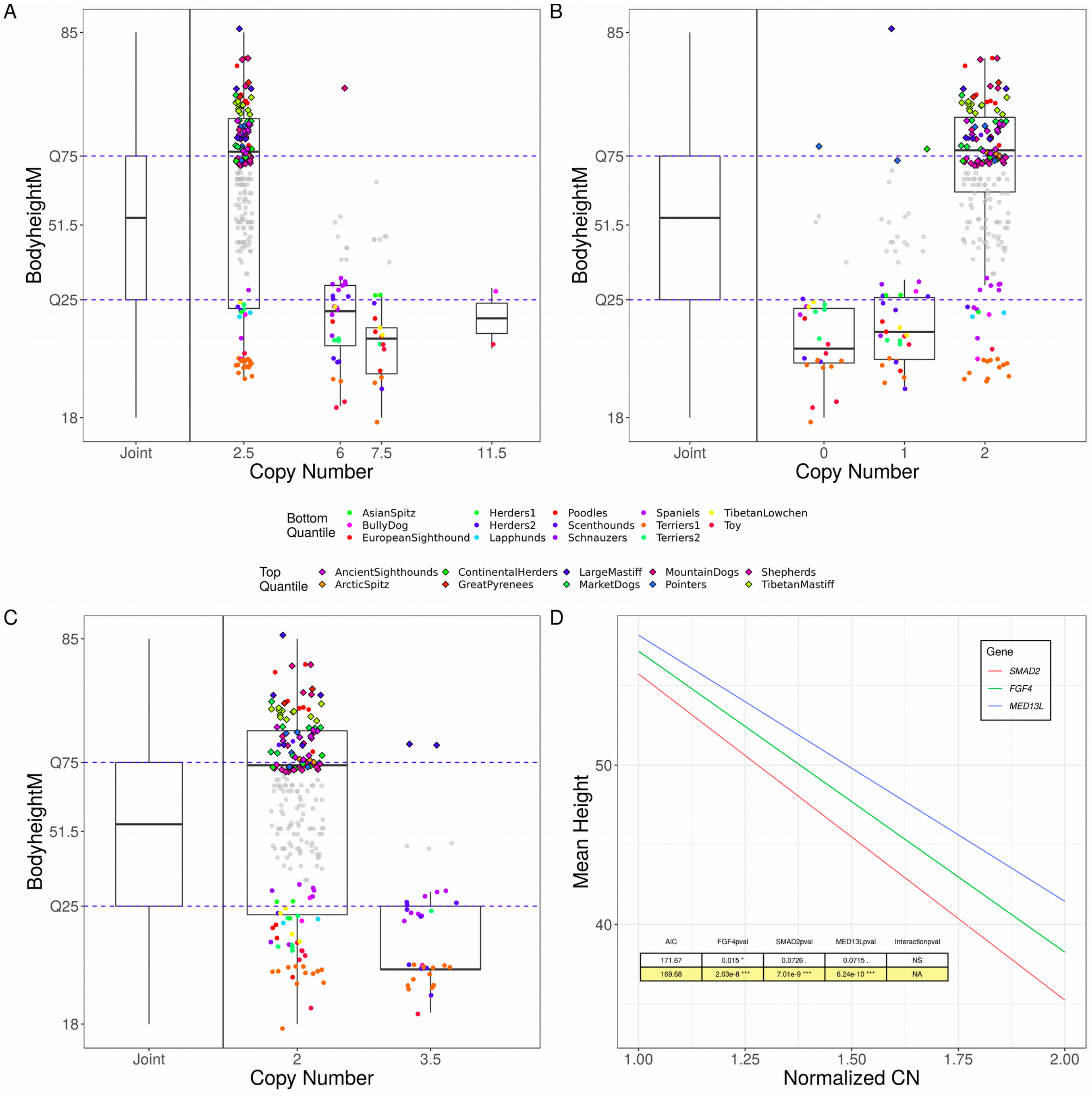

Supplemental Figure S7. (A-C) Boxplot dissection of the three main body mass/height associations *MED13L*, *SMAD2* and *FGF4* respectively. Breeds are colored by trait percentile. Chondrodysplastic breeds were not included in our main body height association analysis to avoid interference of the previously described *FGF4* retrogene (Chr18:48,415,710-48,416,710) with non-syndromic height variation. **(D)** Interaction plot for the three aforementioned genes. Within the plot is a table showing that the model without interaction is preferred.

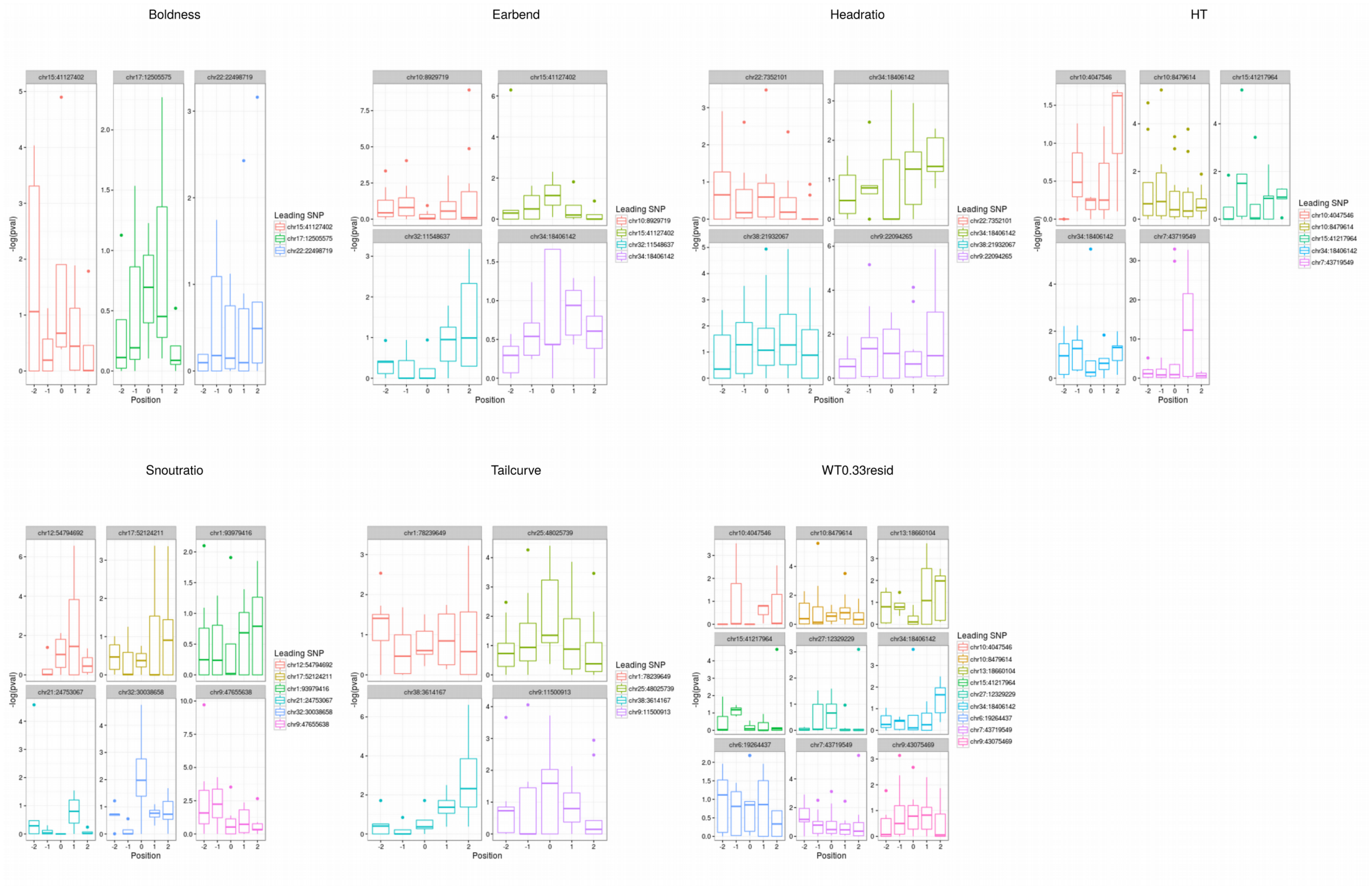

Supplemental Figure S8 – Binned CNV p-values for associated CNVs surrounding leading SNP GWAS results for different traits (methods). The leading SNP hits have been extracted from (Plassais et al. 2019). We detect few instances where the central CNV bins show concordantly small p-values.

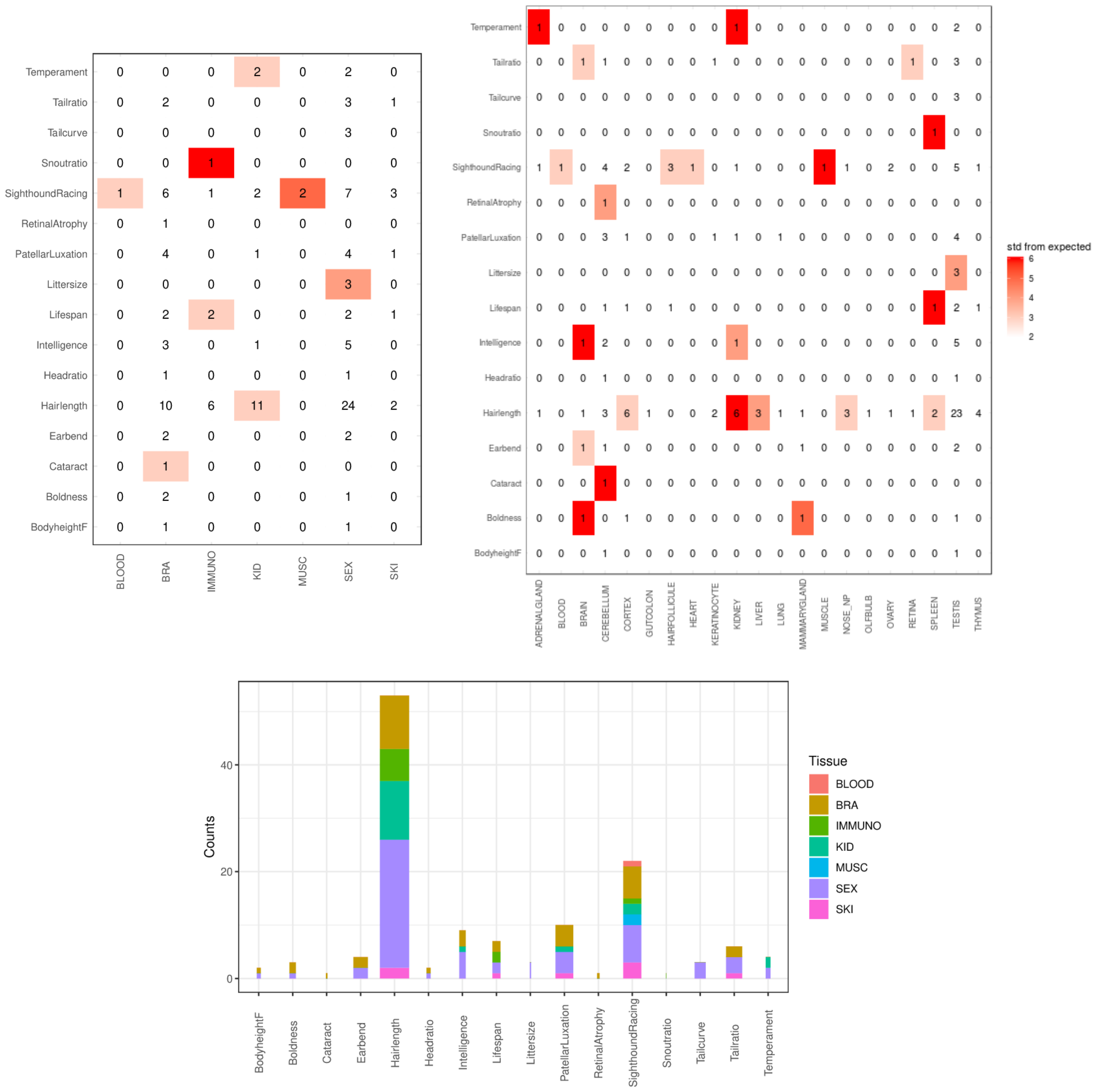

Supplemental Figure S9 – Correlation of lncRNA tissue-specific expression and associated traits. Top: Contingency table of the two variables, the cells with exceeding counts are colored with respect to their deviation from the expected values (methods). Bottom: Alternative visualization of the variables in a stacked barplot of the tissue specific counts. The width of the bars is proportional to the number of associations within the tissue.

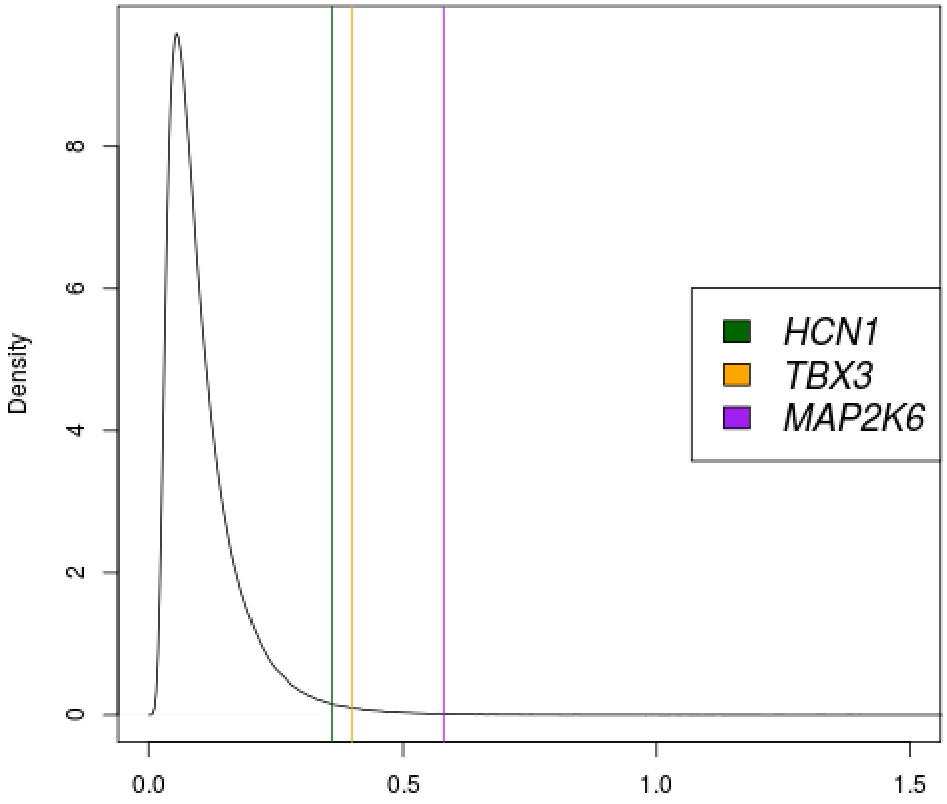

Supplemental Figure S10 – placement of trait-associated Hi-C contacts in the empirical distribution of all normalized CNV contacts (methods). The few associations contained within distal contacts seem to be concordant with the trait they are tagging.
